# *Lactobacillus reuteri* tryptophan metabolism promotes host susceptibility to CNS autoimmunity

**DOI:** 10.1186/s40168-022-01408-7

**Published:** 2022-11-23

**Authors:** Theresa L. Montgomery, Korin Eckstrom, Katarina H. Lile, Sydney Caldwell, Eamonn R. Heney, Karolyn G. Lahue, Angelo D’Alessandro, Matthew J. Wargo, Dimitry N. Krementsov

**Affiliations:** 1 Department of Biomedical and Health Sciences, University of Vermont, Burlington, VT 05401 USA; 2 Department of Microbiology and Molecular Genetics, University of Vermont, Burlington, VT 05401 USA; 3 Department of Biochemistry and Molecular Genetics, University of Colorado, Aurora, CO 80045 USA

**Keywords:** *L. reuteri*, Microbiome, Microbiota, Tryptophan, Multiple sclerosis, Experimental autoimmune encephalomyelitis (EAE), Aryl hydrocarbon receptor (AhR)

## Abstract

**Background:**

Dysregulation of gut microbiota-associated tryptophan metabolism has been observed in patients with multiple sclerosis. However, defining direct mechanistic links between this apparent metabolic rewiring and individual constituents of the gut microbiota remains challenging. We and others have previously shown that colonization with the gut commensal and putative probiotic species, *Lactobacillus reuteri*, unexpectedly enhances host susceptibility to experimental autoimmune encephalomyelitis (EAE), a murine model of multiple sclerosis. To identify underlying mechanisms, we characterized the genome of commensal *L. reuteri* isolates, coupled with in vitro and in vivo metabolomic profiling, modulation of dietary substrates, and gut microbiota manipulation.

**Results:**

The enzymes necessary to metabolize dietary tryptophan into immunomodulatory indole derivatives were enriched in the *L. reuteri* genomes, including *araT*, *fldH*, and *amiE*. Moreover, metabolite profiling of *L. reuteri* monocultures and serum of *L. reuteri*-colonized mice revealed a depletion of kynurenines and production of a wide array of known and novel tryptophan-derived aryl hydrocarbon receptor (AhR) agonists and antagonists, including indole acetate, indole-3-glyoxylic acid, tryptamine, p-cresol, and diverse imidazole derivatives. Functionally, dietary tryptophan was required for *L. reuteri*-dependent EAE exacerbation, while depletion of dietary tryptophan suppressed disease activity and inflammatory T cell responses in the CNS. Mechanistically, *L. reuteri* tryptophan-derived metabolites activated the AhR and enhanced T cell production of IL-17.

**Conclusions:**

Our data suggests that tryptophan metabolism by gut commensals, such as the putative probiotic species *L. reuteri*, can unexpectedly enhance autoimmunity, inducing broad shifts in the metabolome and immunological repertoire.

Video Abstract

**Supplementary Information:**

The online version contains supplementary material available at 10.1186/s40168-022-01408-7.

## Background

Multiple sclerosis (MS) is a chronic autoimmune disease of the central nervous system (CNS) and is the primary cause of non-traumatic neurological disability in young adults [1–3]. Global prevalence and incidence continue to rise, with MS affecting one million people in the USA and more than two million people worldwide [4, 5]. Pathological changes in MS include neuroinflammation, myelin sheath degeneration, axonal damage, and loss of blood-brain barrier integrity [6, 7]. The autoimmune response in MS is characterized by proinflammatory cell CNS infiltration, initiated by Th1/Th17 cells and propagated by innate immune cells and B cells. Resulting clinical features are wide ranging including limb weakness, sensory disturbances, alterations in vision and central nervous system function associated with fatigue, cognitive impairment, depression, and changes to mood, and a myriad of gastrointestinal disturbances [7, 8]. The primary autoimmune animal model of MS, experimental autoimmune encephalomyelitis (EAE), is induced by immunization with myelin antigens, stimulating a myelin-specific Th1/Th17 response that results in CNS inflammatory lesions, demyelination, axonal loss, and neurological dysfunction [9, 10].

The etiology of MS is complex, with both genetic and environmental underpinnings. Environmental risk factors for MS include EBV infection, low vitamin D3 levels, low UV radiation exposure, cigarette smoking, obesity, and potentially diet [5, 11, 12]. A more recently identified putative environmental risk factor for MS is an altered composition of the gut microbiome [13]. The mammalian gut microbiome is a complex ecosystem that consists of bacteria, archaea, viruses, and fungi, totaling > 10 trillion cells, and thousands of microbial species, encoding 100× more genes than does the human genome itself [14, 15]. With regard to MS, the gut microbiome is postulated to impact susceptibility to, or progression of, disease in two major ways: (1) direct interactions between gut bacteria with immune cells, possibly affecting the priming of myelin-reactive T cells, and (2) production of immunomodulatory and/or neuromodulatory catabolites by gut bacteria [13, 16].

Studies of gut microbiota-derived metabolites in MS pathogenesis have predominately focused on microbiota produced short-chain fatty acids (SCFAs), bile acids, and tryptophan metabolites. Abundance of SCFA-producing bacteria [17, 18] and fecal levels of butyrate, propionate, and acetate [17, 19, 20] are lower in MS patients. Further, SCFA supplementation reduces relapse rate and severity of EAE [19, 21–25]. Bile acids are also lower in MS and EAE [26–29], with bile acid supplementation or bile acid receptor modulation ameliorating EAE [27, 30]. Interestingly, tryptophan metabolites, produced by either the host or gut microbiota, have metabolite-specific impact on, and frequently contradictory findings with regard to, MS pathogenesis [31–39].

Mammalian tryptophan metabolism occurs predominately through two major pathways, resulting in the production of serotonin/5-hydroxytryptamine or kynurenine [40]. 5-hydroxytryptamine levels are reduced in the plasma and serum of MS patients [41, 42]. However, treatment with selective serotonin reuptake inhibitors results in only a modest reduction in relapse rate, despite positive findings in EAE models [43–50]. Abundance of kynurenine pathway (KP) metabolites, including kynurenic acid (KA) and quinolinic acid (QA), are altered in MS patients, although the direction of change is not consistent between cohorts [36–38, 51, 52]. Further, the ratio between KA/QA is sufficient to stratify MS patient subtypes, suggesting balance in the KP pathway may be more important than metabolite-specific abundance [39].

Bacterial pathways involved in tryptophan metabolism broadly segregate across the species known to encode them, although the specific enzymes involved, regulatory mechanisms, and impact on the host are often less clear [53, 54]. Tryptophanase (TNA) is historically associated with *E. coli* and functions to convert host dietary tryptophan to indole, which is subsequently converted by the host to indoxyl sulfate [55–57]. Alternatively, tryptophan can be catabolized into tryptamine by tryptophan decarboxylase (TrpD), encoded in a well characterized operon by *Clostridial* species [58]. In a third pathway, tryptophan can be catabolized into a variety of indole derivatives by the aromatic amino acid aminotransferase (ArAT), conserved among some *Lactobacilli* [59–61]. Importantly, substituted indoles generated through the ArAT pathway are described as both elevated or reduced in MS patients, including reports of altered concentrations of indole-3-propionic acid (IPA), indole-3-lactate (ILA), and indole-3-acetate (I3A) [31–33, 62, 63]. These indole derivatives represent known ligands of the immunomodulatory transcription factor, the aryl hydrocarbon receptor (AhR) [64, 65]. Despite altered levels of these putative *Lactobacillus*-produced metabolites in MS patients, 10 of the 14 case-control studies examining the role of the microbiome in MS found no difference in *Lactobacillus* abundance [66–76], with only a single study reporting a clear reduction in *Lactobacillus* abundance in the MS microbiome [77]. The remaining three studies were confounded by lack of taxonomic resolution [28, 78], or reported changes associated with disease-modifying treatment [79]. Collectively, these studies indicate that both mammalian and bacterial tryptophan metabolism are likely important modulators of MS pathogenesis. However, the precise mechanisms of action, causative changes in the gut microbiome, and interplay with the host are unclear.

Animal studies, while more numerous, have also reported divergent findings concerning the role of *Lactobacilli* in modulating EAE pathogenesis, as recently reviewed [80]. Despite this fact, probiotic administration of *Lactobacilli* is being explored as a potential MS therapeutic intervention strategy [81–85]. We and others have recently reported that the stable colonization by the commensal and putative probiotic bacterial species, *Lactobacillus reuteri* (*L. reuteri*), is sufficient to exacerbate EAE pathogenesis in mice [86, 87]. Moreover, we found that the presence of *L. reuteri* was associated with altered levels of circulating tryptophan metabolites [87]. Here, we leveraged whole genome sequencing of commensal *L. reuteri* isolates, which revealed conservation of the enzymatic machinery necessary to catabolize dietary tryptophan into a diverse array of indole derivatives. Consistently, metabolite profiling of *L. reuteri* monocultures revealed production of a broad repertoire of tryptophan derivatives. Functionally, we demonstrate that *L. reuteri* requires dietary tryptophan to exacerbate EAE, which was associated with increased IL-17-producing γδT cell infiltration into the CNS. Additionally, we found that restricting tryptophan availability suppressed CNS autoimmunity in a gut microbiota-dependent fashion. Further, the serum of mice colonized with *L. reuteri* displayed unique dietary tryptophan-dependent metabolic profiles, marked by increased cresols and novel tryptophan-derived imidazoles, and decreased kynurenines. Mechanistically, *L. reuteri*-derived metabolites activated the AhR and enhanced IL-17 production by T cells in vitro. Taken together, our data provide a genetic and metabolic characterization of tryptophan catabolism in a keystone mammalian gut commensal species and establish a species-specific and host diet-dependent mechanism promoting CNS autoimmunity.

## Results

### Isolation and genomic characterization of diverse *Lactobacilli* from murine commensal gut microbiota

In our previous studies, we identified three predominant species of *Lactobacillus* in normal murine gut microbiomes of genetically diverse strains of mice: *L. reuteri*, *L. murinus*, and *L. johnsonii* [87]*.* We note that the *Lactobacillus* genus has been recently reclassified into multiple genera, and these 3 species are now named *Limosilactobacillus reuteri*, *Ligilactobacillus murinus*, and *Lactobacillus johnsonii*, respectively [88]. We opted to refer to them by the original names to maintain consistency with our previous studies. Importantly, we previously demonstrated that the presence of *L. murinus* in the microbiota derived from C57BL/6J (B6) mice was associated with low EAE susceptibility, while the presence of *L. reuteri* derived from the microbiota of genetically distinct wild-derived PWD/PhJ (PWD) mice was associated with high EAE susceptibility, which was functionally confirmed by transfer to B6 mice [87]. To determine if bacterial species-level genetic variation might be responsible for differential capacity to alter EAE pathogenesis, we leveraged whole genome sequencing and assembly of two independent isolates for each species of interest.

**Fig. 1:**
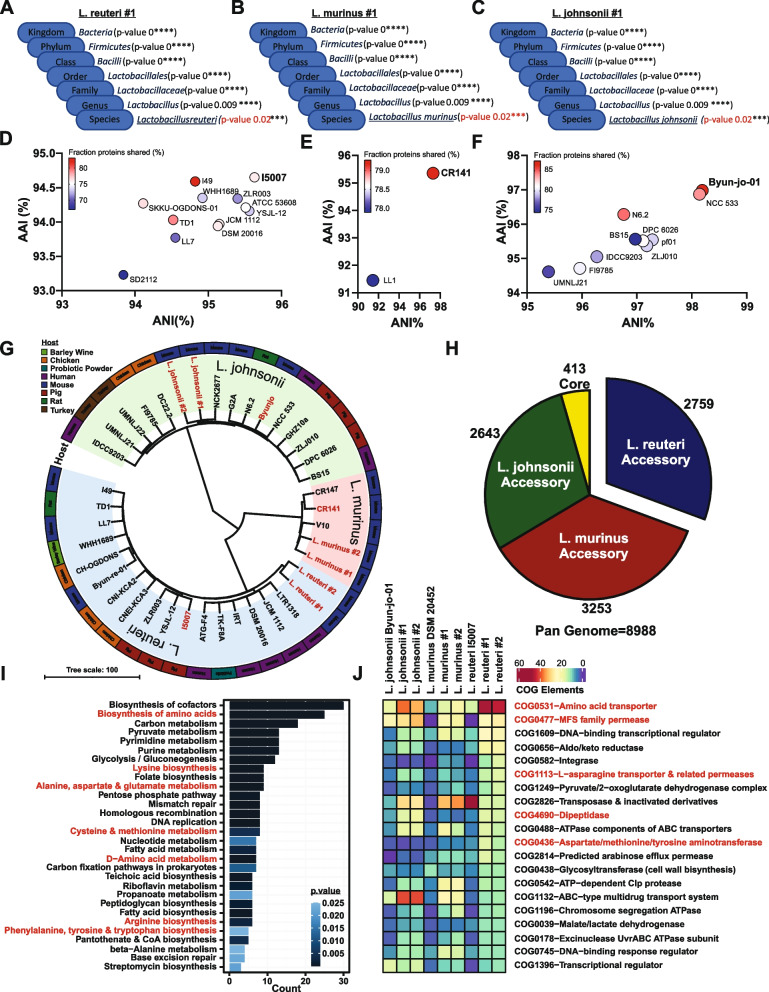
*Lactobacillus* isolates are highly divergent, displaying global differences in metabolic potential. Taxonomic classification of representative draft genomes for *L. reuteri* (**A**), *L. murinus* (**B**), and *L. johnsonii* (**C**) queried against the NCBI non-redundant prokaryotic genomes database with *p*-values representing confidence of phylogenetic assignment. **D**–**F** Nearest subspecies phylogenetic neighbor of each *Lactobacillus* draft genome determined by percent shared genomic content graphed as average nucleotide identity (ANI) verses average amino acid identity (AAI). **G** AAI represented as a phylogenetic tree of *Lactobacillus* isolates and publicly available reference genomes for strains of the same species. Color ranges denote each species with *L. reuteri* in blue, *L. murinus* in red, and *L. johnsonii* in green with direct isolates and nearest subspecies phylogenetic neighbors labeled in red. Outer color strip specifies originating host for each isolate. **H** Pie chart of core and accessory inferred proteomic elements between isolates leveraged for **I** KEGG pathway enrichment analysis of the *L. reuteri* identified accessory genome. (Top 25 enriched pathways at *p*.adjusted<0.05) **J** Heatmap of top 20 clusters of orthologous genes (COG) abundance profiles for *Lactobacillus* isolates and nearest phylogenetic neighbors with warmer colors indicating increasing number of genes assigned to each COG

To confirm species identity and identify the nearest phylogenetic neighbor for each isolate, we queried draft genomes of two independent isolates of each bacterial species against the Microbial Genomes Atlas (MiGA) online server (http://www.microbial-genomes.org) using the NCBI non-redundant prokaryotic genomes database [89]. Species-level identity for each isolate was confirmed at *p*<0.05, with the exception of *L. reuteri* isolate #1 at *p*<0.08 (Fig. 1A–C and Table S1). Both average nucleotide identity (ANI) and average amino acid identity (AAI), as percent identity and fraction of genome shared, were analyzed to determine nearest phylogenetic neighbors between closely related and distant taxa respectively. Within each species, each of the two isolates was most closely related to the same subspecies, as indicated by both metrics. Draft genomes of *L. reuteri*, *L. murinus*, and *L. johnsonii* isolates were most closely related to *L. reuteri subspecies I5007*, *L. murinus subspecies CR141*, and *L. johnsonii subspecies Byun-jo-01* respectively (Fig. 1D–F, Table S2 and Fig. S1).

To broadly determine the extent of genomic and inferred proteomic variation between our isolates and bacterial strains of the same species, available complete high-quality draft genomes, including MIGA-identified nearest phylogenetic neighbors, were analyzed together with our isolate draft genomes using the ANI/AAI-Matrix calculator [90]. AAI represented as a phylogenetic tree revealed distinct interspecies clades. Notably, isolates clustered most closely with strains of human and mouse origin, with *L. reuteri* and *L. murinus* more similar to each other than either species is to *L. johnsonii* (Fig. 1G). To infer mechanistic potential of divergent loci unique to *L. reuteri*, protein coding genes were segregated into core and accessory content using the Bacterial Pan Genome Analysis tool (BPGA) and mapped to both KEGG & COG databases. Interspecies clustering using BPGA identified a high percent of accessory inferred proteomic content, ranging from 86.3 to 88.7% accessory, with 11.3–13.7% core proteome totaling only 400 genes (Fig. 1H and Table S3). To determine specifically which elements were enriched in the *L. reuteri* genome, core, accessory, and unique genomic KEGG orthology identifiers were extracted for KEGG enrichment analysis using clusterProfiler [91]. The *L. reuteri* accessory genome was found to be enriched in amino acid metabolism and biosynthesis (Fig. 1I and Tables S4-S6). Differentially abundant COG elements within the *L. reuteri* genome also indicated enrichment of loci involved in amino acid transport and breakdown, including transporters, permeases, dipeptidases, and aminotransferases potentially involved in processing of tryptophan (Fig. 1J and Table S7). Taken together, these data suggest global differences in metabolic potential within *Lactobacillus* isolates, with enhanced utilization of amino acids within *L. reuteri* isolates.

### The *L. reuteri* genome is enriched in genes involved in amino acid production and utilization, including catabolism of tryptophan

Our findings above indicated that the *L. reuteri* genomes may be enriched in genes responsible for tryptophan metabolism, which is consistent with the known conservation of the machinery necessary to catabolize host dietary tryptophan into indole derivatives among *Lactobacilli* [59–61]. To determine if our *L. reuteri* isolates encode the enzymes necessary to perform this function, we compared the presence/absence and number of the key known loci essential for tryptophan utilization encoded in the genomes of our *Lactobacilli* to that in the (1) 182 *Lactobacillus* representative genomes available on PATRIC, (2) high-quality complete reference genomes for each species, and (3) the nearest phylogenetic neighbors of each isolate.

**Fig. 2:**
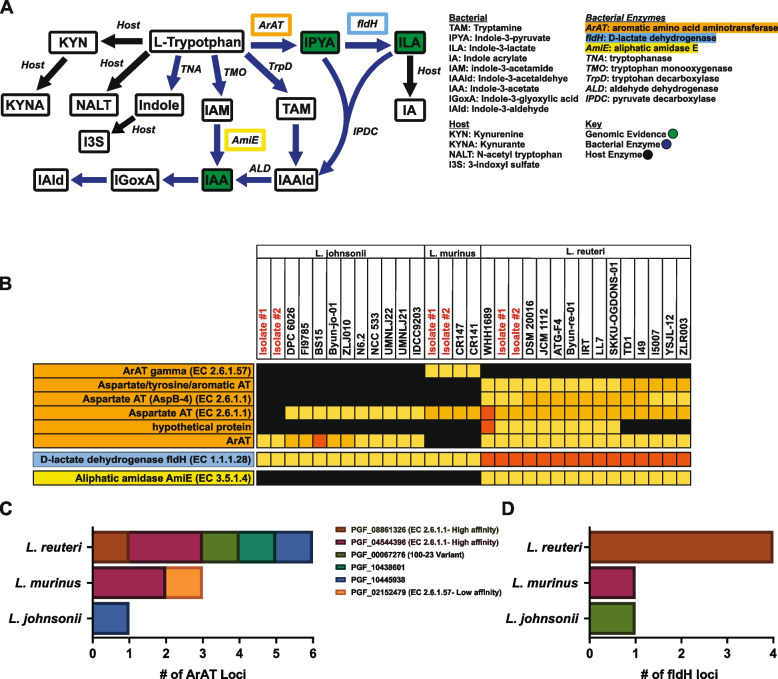
The *L. reuteri* genome is enriched in genes involved in amino acid production and utilization, including catabolism of tryptophan. **A** Pathway schematic of bacterial and abbreviated mammalian tryptophan metabolism. Enzymes with genomic evidence in *Lactobacillus* isolate are boxed in orange (ArAT), blue (FldH), and yellow (AmiE). **B** Heatmap of bacterial tryptophan specific enzymes with genomic evidence in *Lactobacillus* isolates. Enzymes are listed along the left in corresponding colors to the pathway in **A** with isolates and representative strains of the same species along the top and warmer colors indicating increasing copy number. Gene count colored by class of each ArAT or FldH is graphed in **C** and **D** respectively

In bacteria, four main enzymes within the “indole” pathway directly utilize tryptophan as substrate including tryptophanase (TNA), tryptophan monooxygenase (TMO), tryptophan decarboxylase (TrpD), and aromatic amino acid aminotransferases (ArAT) (Fig. 2A). We found that all *Lactobacillus* reference genomes and our isolates do not encode a clear ortholog of TNA, TMO, or TrpD. However, the loci encoding ArATs, which convert tryptophan into indole-3-pyruvate, were broadly conserved among *Lactobacillus* species, as a wide array of enzyme classes (Tables S8-S14), known to have differing affinity for tryptophan as substrate [92]. Each of our isolates was broadly similar in both number and class of aminotransferase as compared to their nearest phylogenetic neighbors, with a greater number and variety encoded within the genome of *L. reuteri* (Fig. 2B, C and Tables S11-S14). Further, *L. reuteri* isolates were enriched in tryptophan high affinity classes of ArAT (EC 2.6.1.1), while *L. murinus* was the sole species encoding the lower affinity variant (E.C. 2.6.1.57) of this enzyme (Fig. 2C and Table S10). To assess mRNA expression profiles of predicted *araT* loci, *L. reuteri* was cultured in brain heart infusion medium (BHI) with or without 1 mM tryptophan supplementation, for 4 or 24 h, for analysis by qRT-PCR. 1mM tryptophan supplementation was selected as a physiologically relevant concentration similar to what *L. reuteri* would be exposed to within the small intestine [93–95]. By 4 h of monoculture, robust expression was observed, with a tryptophan responsive increase in 5 of 6 putative *araT*genes, which was sustained at the 24 h timepoint (Fig. S2C and D). Although variation in the level of expression and degree of response to tryptophan was observed, these data indicate intact functional loci as genomically predicated.

To our knowledge, the majority of the other enzymes involved in bacterial tryptophan metabolism (Fig. 2A) have not been previously characterized in detail for *Lactobacilli*. We failed to find genomic evidence for the presence of indole acetaldehyde dehydrogenase (ALD) and indole-3-pyruvate decarboxylase (IPDC) in our isolates, with spotty conservation among *Lactobacillus* reference genomes (Table S15). However, we did find evidence of conservation of phenyllactate dehydrogenase (*fldH*) and indole acetamide hydrolase (aliphatic amidase E/*amiE*) (Fig. 2B, D and Tables S16 and S17). FldH acts on indole-3-pyruvate produced by ArAT to form indolelactate (Fig. 2A). While the specific *fldH* gene previously described in *Clostridia* [96] was not annotated in *Lactobacilli*, lactate dehydrogenases with the same enzyme commission number and cross-genus global family designation were present and enriched in *L. reuteri* isolates as compared to *L. murinus* or *L. johnsonii* isolates and reference strains (Fig. 2B, D). Importantly, *L. reuteri* was the only genome in which *fldH* was found directly adjacent to *araT* (Fig. S3), suggesting the presence of a functional metabolic gene cluster/operon in *L. reuteri* that is not intact in the other two species. Further, BLAST analysis with the original FldH enzyme identified and characterized in *Clostridial* species [96], revealed high sequence conservation with *fldH* encoded by *L. reuteri* isolates at ~98% coverage and ~40% amino acid sequence homology (Table S16). Lastly, *L. reuteri* was also the only isolate encoding AmiE (Fig. 2B, Tables S18 and S19), functioning to convert the non-AhR agonist indole-3-acetamide into indole-3-acetic acid, a known AhR ligand. Importantly, the presence of *amiE* was surprising given the lack of genomic evidence for conservation of TMO yielding the upstream substrate for this enzyme, indole-3-acetamide. Taken together, these data suggest that, compared with other *Lactobacilli*, the *L. reuteri* genomes, including those of our isolates, encode a greater number and variety of genes necessary to metabolize host dietary tryptophan into indole derivatives.

### *L. reuteri* produces a wide array of tryptophan-derived metabolites

Based on the genomic evidence that *L. reuteri* could produce a wide array of tryptophan-derived catabolites, we sought to directly assess the repertoire of metabolites produced by *L. reuteri* in vitro in response to modulation of tryptophan availability. Monocultures of *L. reuteri* were grown under anaerobic conditions in BHI medium with or without 1 mM tryptophan supplementation, for metabolic profiling via ultrahigh-performance liquid chromatography-tandem mass spectroscopy (UPLC-MS/MS) (Table S20) [97]. Partial least squares-discriminate analysis (PLS-DA) and hierarchical clustering by Euclidean distance of total metabolites from basal BHI medium and *L. reuteri* monocultures with or without 1 mM tryptophan supplementation, separated the samples into discrete clusters, indicating differential metabolite production by *L. reuteri* in response to tryptophan availability (Fig. S4A, B and Table S21).

**Fig. 3:**
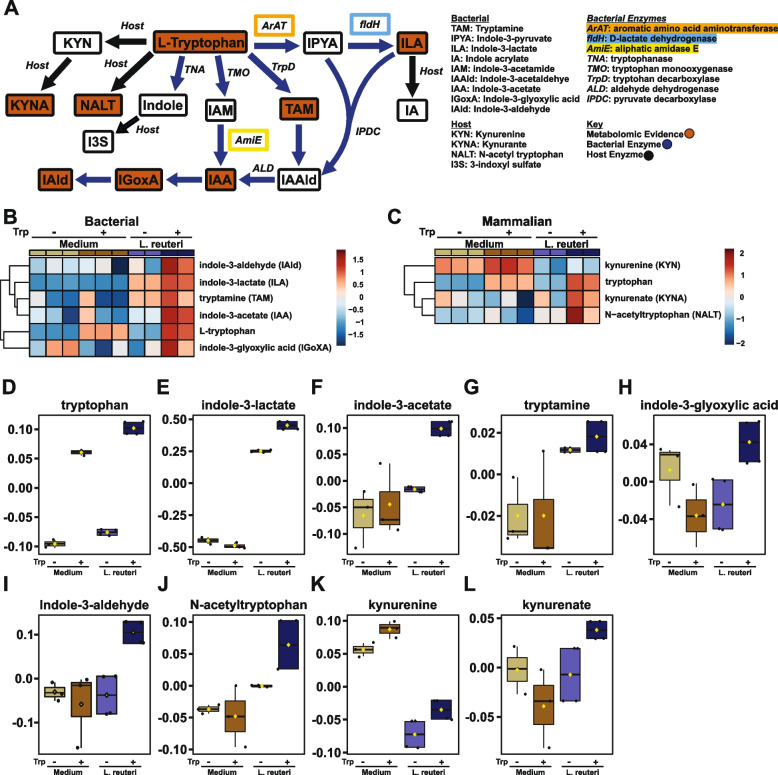
*L. reuteri* produces a wide array of tryptophan-derived metabolites known to modulate the immune system. **A** Pathway schematic of bacterial and abbreviated mammalian tryptophan metabolism. Enzymes with genomic evidence in *Lactobacillus* isolates are boxed in orange (ArAT), blue (FldH), and yellow (AmiE) with metabolites produced in *L. reuteri* monoculture in red. Heatmaps of bacterial (**B**) and mammalian (**C**) tryptophan metabolites significantly modulated by *L. reuteri* in monoculture (*n*=2 per group) (*p*<0.05) as compared to medium alone controls (*n*=3 per group) with or without 1mM tryptophan supplementation where warmer colors indicate increased relative abundance. Corresponding abundance profiles are shown in **D**–**L**

To directly assess the repertoire of *L. reuteri*-produced tryptophan catabolites, we restricted our analyses specifically to tryptophan pathway-associated mammalian and bacterial products (Fig. 3A). Based on genomic evidence for the presence of loci encoding the ArAT enzyme (Fig. 2B, C), as the first and rate limiting step in *Lactobacillus*-specific tryptophan metabolism, we expected to observe a tryptophan-dependent increase in indole-3-pyruvate (Fig. 3B). Indole-3-pyruvate was not detected in any of our samples, perhaps because this metabolite is quickly shunted into two major branches of the indole pathway (Fig. 3A) producing either indole-3-lactate or indole-3-acetaldehyde. Consistent with this notion and with the presence of genes encoding D-lactate dehydrogenase (Fig. 2B and D), *L. reuteri*-conditioned medium contained a high abundance of indole-3-lactate, with further elevation upon tryptophan supplementation (Fig. 3B, E and Table S22). Similarly, consistent with conservation of the AmiE enzyme in the *L. reuteri* genome (Fig. 2B), there was a tryptophan-dependent increase in indole-3-acetate abundance (Fig. 3B, F and Table S22). Surprisingly, *L. reuteri* also produced tryptamine at appreciable levels compared to basal medium (Fig. 3B, G and Table S22). While we could not confidently annotate the decarboxylase responsible for catabolizing tryptophan into tryptamine [58], we found one enzyme within the genome of *L. reuteri*, with ~40% sequence homology to the known *Clostridial* identified decarboxylase that performs this function (Tables S16 and S17). Further, we also observed an *L. reuteri* tryptophan-dependent production of indole-3-glyoxylic acid and indole-3-aldehyde (Fig. 3A, B, H, I and Table S22), the former of which has not been previously described in *Lactobacilli*, despite evidence in other genera [98]. Surprisingly, the presence of *L. reuteri* also slightly elevated the level of tryptophan (Fig. 3B, D and Table S22), suggesting that in addition to tryptophan catabolism, this species can readily synthesize this amino acid de novo.

While “classically mammalian” tryptophan metabolites were not expected to be produced or altered in the context of *L. reuteri* monoculture, several were altered compared to the basal medium alone. *L. reuteri* cultures were marked by accumulation of N-acetyl tryptophan (Fig. 3C, J and Table S22). Further, while kynurenine was present at appreciable levels in basal medium, *L. reuteri* appeared to actively deplete this metabolite (Fig. 3C and K and Table S22). In parallel, while basal media did not contain appreciable levels of kynurenate, *L. reuteri* cultures accumulated kynurenate in a tryptophan-dependent manner (Fig. 3C, L and Table S22). Together with genomic data, our metabolomic analysis demonstrates that *L. reuteri* can produce a diverse profile of both novel and known tryptophan-derived metabolites and suggests that this bacterium may compete with the host kynurenine pathway.

### Tryptophan availability modulates CNS autoimmunity in a microbiota-dependent fashion

**Fig. 4:**
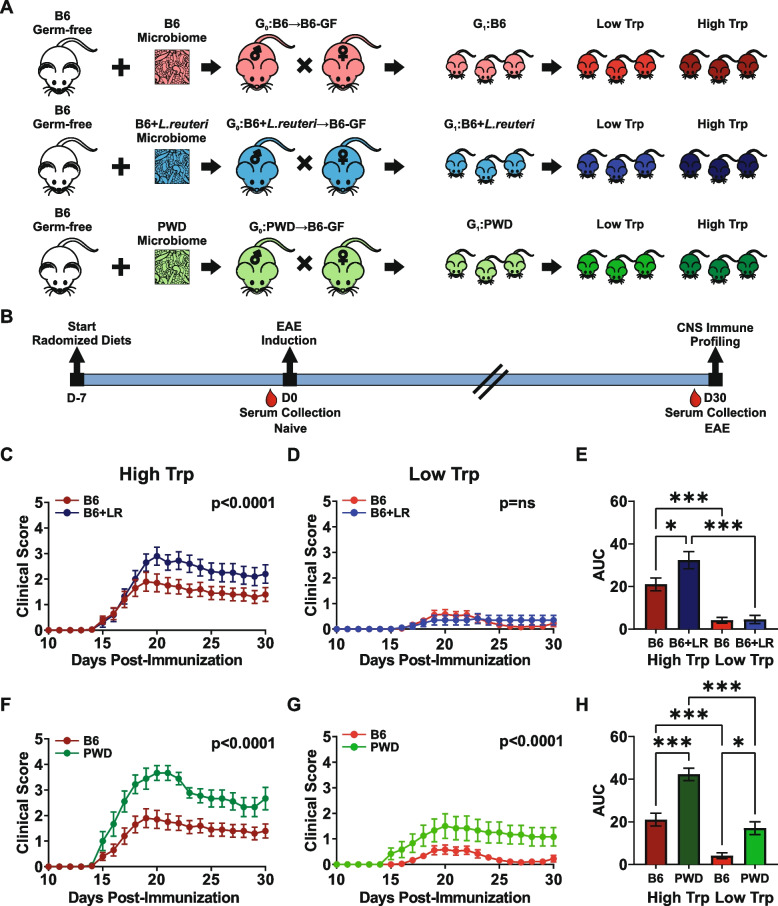
Tryptophan availability modulates CNS autoimmunity in a microbiota-dependent fashion. **A** Schematic of microbiome transplantation and dietary tryptophan modulation model. Ex-germ-free mice received either B6, B6+*L. reuteri*, or PWD cecal microbiota transplantation, denoted B6→B6-GF (*n*=38), B6+*L. reuteri*→B6-GF (*n*=37), or PWD→B6-GF (*n*=22), serving as G_0_ breeding pairs for the vertical transmission model. Offspring were randomized to either a low (0.02%) or high (0.8%) tryptophan diet 1 week prior to EAE induction and serum was collected from naïve and post-EAE mice (**B**). **C**–**H** EAE was evaluated in ex-GF GMT recipients reflected as mean daily clinical score, with overall significance determined by Friedman’s non-parametric two-way ANOVA and one-way ANOVA of area under the curve (AUC)

To determine if the observed genomic and metabolomic enrichment in the capacity to utilize tryptophan (Figs. 2 and 3) is directly responsible for *L. reuteri*-dependent exacerbation of CNS autoimmunity, we leveraged our previously established gut microbiota transplantation and vertical transmission model [87]. In this model, we generated genetically identical murine hosts colonized with three distinct microbiota configurations, containing or lacking *Lactobacillus* species of interest; coupled with manipulation of dietary tryptophan levels (summarized schematically in Fig. 4A, B). Specifically, germ-free (GF) B6 mice were colonized with (1) B6 cecal microbiota (B6→B6-GF, naturally harboring *L. murinus* and *L. johnsonii*), (2) B6 cecal microbiota supplemented with 10^9^ CFU *L. reuteri* (B6+*L.reuteri*→B6-GF) which we previously demonstrated exacerbates EAE compared to B6 alone, (3) cryopreserved PWD cecal microbiota (PWD→B6-GF, naturally harboring *L. reuteri* and *L. johnsonii*), which had also exacerbated EAE compared with B6 microbiota [87]. Colonized ex-GF mice were used to establish G_0_ (generation zero) breeding pairs, which vertically passed their microbiota to their offspring (G_1_). Resulting G_1_ offspring were randomized to either a 0.02% (low) or 0.8% (high) tryptophan diet 1 week prior to induction of EAE and maintained on these diets continuously until the end of experimentation. EAE was induced via immunization with MOG_35-55_ as previously described [87]. In the presence of high levels of dietary tryptophan, the introduction of *L. reuteri* into B6 microbiota exacerbated EAE compared with B6 baseline microbiota (Fig. 4C, E), recapitulating our previous findings in the context of a normal tryptophan-replete diet [87]. Importantly, dietary tryptophan restriction abrogated the ability of *L. reuteri* to exacerbate EAE, as no difference in EAE severity was observed between mice harboring B6 microbiota and B6+*L. reuteri* microbiota on the low-tryptophan diet, with a near complete suppression of EAE across both microbiota configurations (Fig. 4D, E). In contrast, mice harboring PWD microbiota showed significantly higher EAE severity than mice harboring B6 microbiota in the presence of high or low dietary tryptophan (Fig. 4F–H), suggesting that the PWD microbiota can augment EAE independent of tryptophan. Measurement of *L. reuteri* abundance in fecal samples revealed that while *L. reuteri* abundance across individual *L. reuteri*-*c*olonized mice did not correlate strongly with disease severity, mice fed a high-tryptophan diet maintained a higher abundance of *L. reuteri* through the course of dietary intervention (Fig. S5). Together, these data show that depletion of tryptophan suppresses CNS autoimmunity, and for the first time that tryptophan-dependent EAE pathogenesis can be causally linked to *L. reuteri*.

### Tryptophan alters CNS-infiltrating immune cell populations via distinct diet- and microbiota-dependent mechanisms

**Fig. 5:**
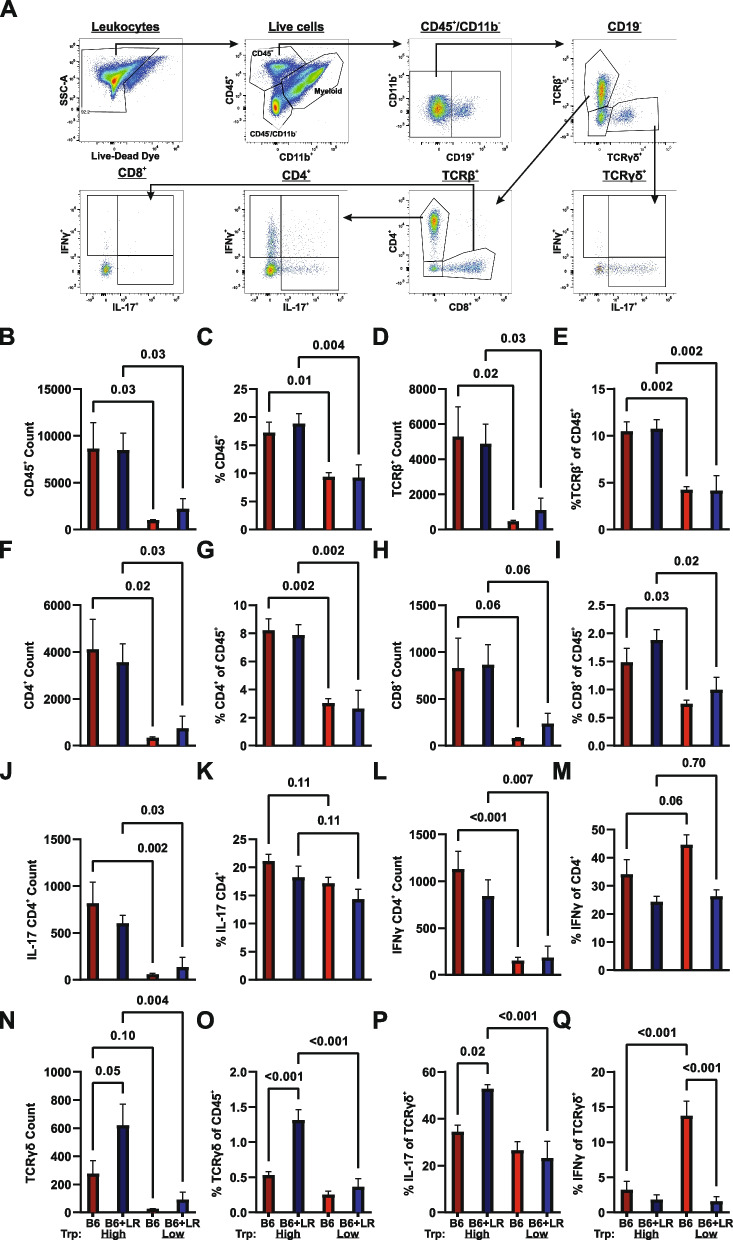
Tryptophan alters CNS-infiltrating immune cell populations via distinct diet and microbiome-dependent mechanisms. CNS-infiltrating leukocytes were isolated and analyzed by flow cytometry (**A**) 30 days post-EAE induction in ex-GF GMT recipients harboring the B6 or B6+*L. reuteri* microbiota randomized to a low- or high-tryptophan diet are in (Fig. 4C–E). Major effects of diet alone including (**B**, **C**) count and frequencies of the total CD45^+^HighCD11b^−^ population, **D**, **E** TCRb^+^, **F**, **G** CD4^+^, **H**, **I** CD8^+^ T cells, **J**, **K** CD4^+^ IL17 and **L**, **M** IFNγ . *L. reuteri* and tryptophan-dependent immunological response in TCRγδ T cells (**N**, **O**) and cells positive for indicated cytokines (**P**, **Q**)

To determine the cellular mechanisms associated with *L. reuteri-* and tryptophan-dependent EAE exacerbation, we analyzed by flow cytometry immune cells infiltrating into the spinal cord of diseased mice harboring B6 microbiota or B6+*L. reuteri* microbiota on tryptophan low or high diets. Phenotypic changes resulting from dietary intervention fell within two categories: *L. reuteri-*dependent or *L. reuteri*-independent. *L. reuteri*-independent changes included a striking decrease in the proportion and number of total CD45^+^CD11b^−^ leukocytes, as well as TCRβ^+^, CD4^+^, and CD8^+^ T cells in the spinal cord in response to tryptophan depletion, irrespective of the presence of *L. reuteri* (Fig. 5B–I). The number of IL-17 and IFNγ-producing CD4^+^ T cells was also reduced with low tryptophan, although the frequency of CD4^+^ T cells producing cytokines was not significantly affected (Fig. 5J–M). In contrast, *L. reuteri* colonization elevated the number and frequency of TCRγδ cells and their production of IL-17, in the presence of high dietary tryptophan (Fig. 5N–P). In contrast, IFNγ production by TCRγδ cells followed an inverse trend (Fig. 5Q). Taken together, these data suggest that while restriction of dietary tryptophan globally decreases encephalitogenic lymphocyte accumulation in the CNS, correlating with suppressed EAE symptomology, diets replete with tryptophan can enhance CNS autoimmunity in a microbiota-dependent manner through divergent cellular mechanisms, potentially involving non-conventional T cell subsets.

### Dietary tryptophan availability profoundly alters systemic circulating host and bacterial metabolites

**Fig. 6:**
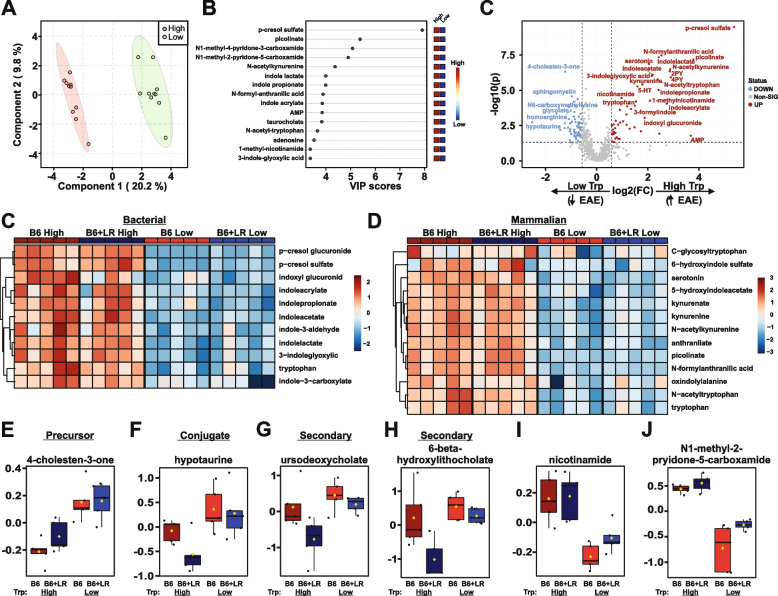
Dietary tryptophan availability alters systemic circulating host and bacterial metabolites. Serum was collected from mice fed a low- or high-tryptophan diet colonized with either B6 or B6+*L. reuteri* microbiota following a 30-day EAE course and analyzed via UPLC-MS/MS as outlined in Fig. 4A, B. Data were analyzed independent of the microbiota to highlight effect of diet alone. **A** Partial least squares-discriminant analysis (PLS-DA) of total metabolites. **B** Top 15 metabolites as variables of importance in the PLS-DA projection (VIP) along component 1. **C** Volcano plot of differentially abundant metabolites, passing a threshold of |FoldChange|>1.5 and *P*<0.05. Positive fold-change indicates higher abundance in high-tryptophan-fed mice exhibiting higher EAE severity (Fig. 4). Heatmaps of differentially abundant bacterial (**C**) and mammalian (**D**) tryptophan-associated metabolites analyzed one-way ANOVA. Post hoc analysis using Fisher’s LSD of selected bile acids (**E**–**H**) and nicotinamide associated (**I**, **J**) are represented as log-transformed and mean centered abundance plots

To determine the global impact of tryptophan restriction on systemic metabolic profiles in vivo, serum was collected from mice fed either the low- or high-tryptophan diet following a full 30-day EAE disease course and analyzed via UPLC-MS/MS (Fig. 4B, Tables S23 and S24 and Fig. S6). Data were pooled independent of microbiota (B6 and B6+*L. reuteri*) to specifically analyze changes driven by diet alone. PLS-DA analysis revealed clear segregation by diet along component 1, capturing approximately 20% of the total variance (Fig. 6A). Extracting the top 15 metabolites as variables of importance (VIP) in the PLS-DA projection along component 1 (Table S25) revealed the expected tryptophan-associated metabolites, including a number of bacterial indoles produced by *L. reuteri* in monoculture (Figs. 3B–L and 6B). Further, a number of classically mammalian tryptophan metabolites, including N-acetyl-kynurenine and N-acetyl-tryptophan (Fig. 6B) and a striking tryptophan-dependent increase in p-cresol sulfate, were observed. Similar results were obtained by direct fold change analysis between mice fed low- and high-tryptophan diets, represented as a volcano plot (Fig. 6C and Table S26) or as heatmaps (Fig. 6C, D and Tables S27 and S28). In addition to alteration of systemic tryptophan-associated metabolites, mice fed a low-tryptophan diet had an increase in bile acid-associated metabolites including 4-cholesten-3-one, hypotaurine, ursedeoxycholate, and 6-beta-hydroxylithocholate (Figs. 6E–H and 8B). Of note, conjugated and secondary bile acid increase in low-tryptophan-fed mice appeared to be *L. reuteri* dependent (Fig. 6F–H). Further, mice fed a low-tryptophan diet were also depleted in circulating nicotinamides including N1-methyl-4-pyridone-3-carboxamide, N1-methyl-2-pyridone-5-carboximide, and nicotinamide itself (Fig. 6C, I, J and Table S26), consistent with the role of tryptophan as the precursor to nicotinamide synthesis [99]. Taken together, low-tryptophan-diet-mediated suppression of EAE is marked by a stark decrease in both bacterial and mammalian tryptophan metabolites with a reduction in nicotinamide metabolism and an increase in bile acids.

### Aromatic cresols are markers of *L. reuteri* tryptophan-dependent enhanced autoimmune predisposition

**Fig. 7:**
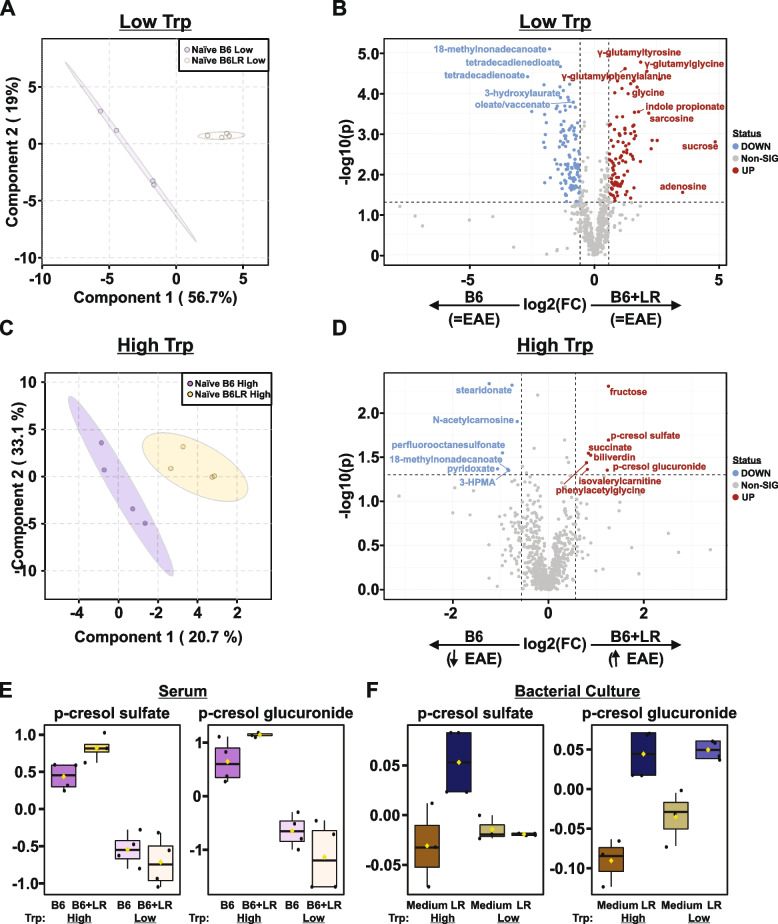
Aromatic cresols are markers of *L. reuteri* tryptophan-dependent enhanced autoimmune predisposition. Serum was collected from naïve mice fed a low- or high-tryptophan diet colonized with either the B6 or B6+*L. reuteri* microbiota and analyzed via UPLC-MS/MS as outlined in Fig. 4A, B. Partial least squares-discriminant analysis (PLS-DA) and volcano plots of differentially abundant metabolites, passing a threshold of |FoldChange|>1.5 and *P*<0.05 in low-tryptophan-fed mice (**A**, **B**) or high-tryptophan-fed mice (**C**, **D**). Post hoc analysis using Fisher’s LSD of aromatic cresols in serum (**E**) or *L. reuteri* monoculture (**F**) are represented as log-transformed and mean centered abundance plots

**Fig. 8:**
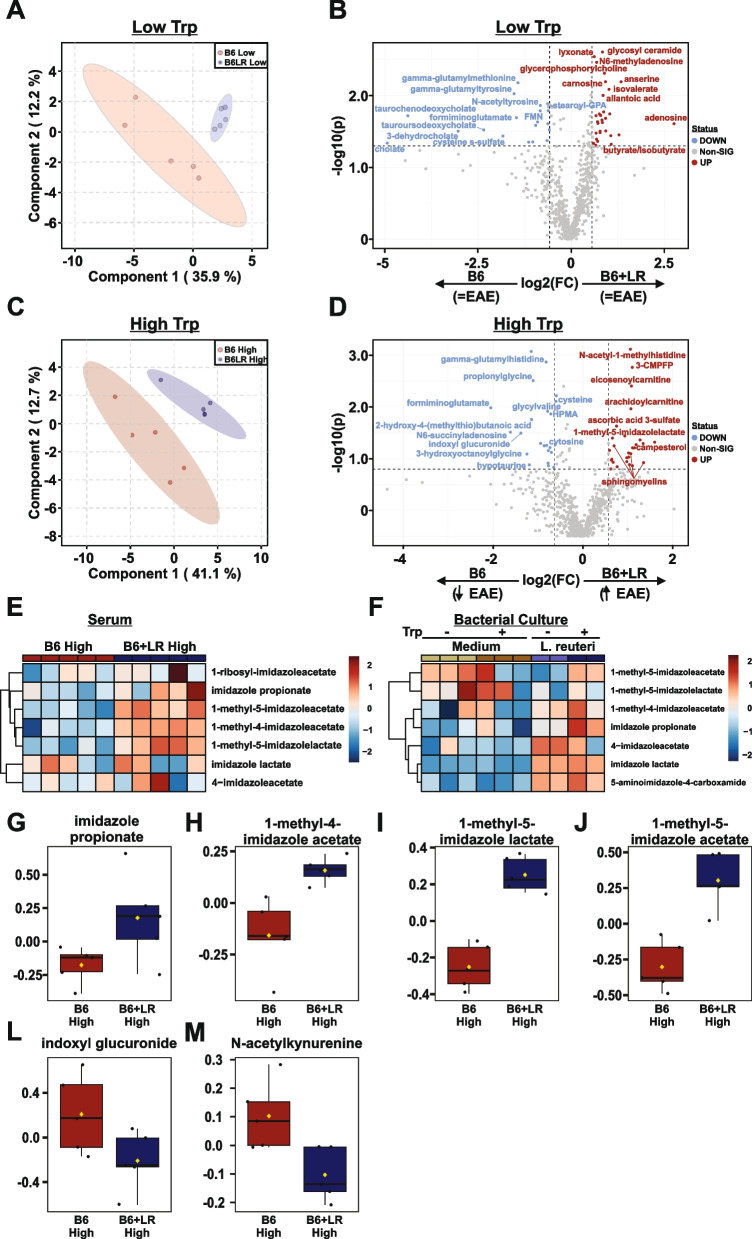
An imbalance between mammalian and novel *L. reuteri* tryptophan metabolites is associated with enhanced autoimmunity. Serum was collected from mice fed a low- or high-tryptophan diet colonized with either the B6 or B6+*L. reuteri* microbiota following a 30-day EAE course and analyzed via UPLC-MS/MS as outlined in Fig. 4A, B. Partial least squares-discriminant analysis (PLS-DA) and volcano plots of differentially abundant metabolites, passing a threshold of |FoldChange|>1.5 and *P*<0.05 in low-tryptophan-fed mice (**A**, **B**) or high-tryptophan-fed mice (**C**, **D**). Heat maps of differentially abundant imidazoles in the serum of high-tryptophan-fed mice (**E**) or *L. reuteri* monoculture (**F**). Post hoc analysis using Fisher’s LSD of serum imidazoles (**G**–**J**), indoxyl glucuronide (**L**), and N-acetyl kynurenine (**M**) are represented as log-transformed and mean centered abundance plots

We next sought to understand how *L. reuteri*-driven metabolic changes might lead to autoimmune predisposition in the presence of dietary tryptophan (Fig. 4C–E). To begin to answer this question, we used UPLC-MS/MS to analyze serum metabolites from mice colonized with the B6 control microbiota or B6+*L.reuteri* microbiota at two distinct time points: (1) following 1 week of dietary intervention, just prior to EAE induction (naïve; Fig. 7), and (2) at 30 days post-EAE induction and 5 weeks total of diet (Fig. 8). For naïve mice, PLS-DA analysis revealed sample clustering based on the presence of *L. reuteri*, on both low- and high-tryptophan diets (Fig. 7A, C). However, the presence of *L. reuteri* was associated with a distinct metabolic profile depending on diet (Fig. 7B, D, Tables S29 and S30). Notably, even under conditions of tryptophan restriction, the presence of *L. reuteri* was sufficient to elevate levels of indole propionate (Fig. 7B). Consistent with *L. reuteri*’s high genomic potential for amino acid metabolism, including enrichment in bacterial dipeptidases and transpeptidases (Fig. 1J and Table S7), the presence of *L. reuteri* was also marked by elevated glutamine-containing dipeptides, with a reduction in unsaturated long-chain fatty acids (Fig. 7B). Importantly, with increased tryptophan availability, *L. reuteri* colonization resulted in marked elevation of the cresol containing metabolites, p-cresol sulfate and p-cresol glucuronide (Fig. 7D–F). Interestingly, the abundance of both cresols also correlated with disease severity, represented as cumulative disease score, the sum of all daily EAE scores over 30 days (Fig. S7 and Table S31). Given their neurotoxic nature and recent reports finding elevated levels within the cerebrospinal fluid of MS patients, these bacterial-derived metabolites were of particular note [100]. To determine if *L. reuteri* itself can produce cresols, we leveraged our bacterial monoculture data to specifically assess their presence. Indeed, we found that *L. reuteri* produced both p-cresol sulfate and p-cresol glucuronide, with the former accumulating in a tryptophan-dependent manner (Fig. 7F). Taken together, *L. reuteri* modulates levels of circulating metabolites in the naïve mouse, with a tryptophan-dependent increase in cresol metabolites as markers of subsequent enhanced disease pathology.

### An imbalance between mammalian and novel *L. reuteri* tryptophan metabolites is associated with enhanced autoimmunity

We next assessed the metabolic effect of *L. reuteri* colonization following a full 30-day EAE course in mice fed a high- or low-tryptophan diet for a total of 5 weeks, to identify metabolic changes associated with tryptophan-dependent exacerbation of EAE by *L. reuteri*. Despite similar EAE severity between *L. reuteri*-colonized and control mice in the absence of dietary tryptophan (Fig. 4C), mice fed a low-tryptophan diet segregated into unique microbiota-dependent clusters based on the levels of circulating metabolites (Fig. 8A). Fold change analysis revealed a decrease in glutamate dipeptides and in the bile acids taurochenodeoxycholate, tauroursodeoxycholate, and cholate, echoing the global effect of tryptophan depletion (Fig. 6B, C), with an increase in the SCFAs, butyrate and isovalerate (Fig. 8B) in *L. reuteri*-replete microbiota (Table S32).

Similar to low-tryptophan diets, clustering based on the level of circulating metabolites in samples from high-tryptophan-fed mice segregated distinctly by the presence of *L. reuteri* (Fig. 8C). In high-tryptophan-fed mice, *L. reuteri* drove a reduction in histidine-associated metabolites, including formiminoglutamate and gamma-glutamylhistidine, as well as tryptophan metabolites indoxyl glucuronide, and acetylated kynurenine, with an increase in carnitine-conjugated long-chain fatty acids and sphingomyelin or sphingosine containing metabolites (Fig. 8C and L–M). Reduction of the liver metabolite of bacterial TNA-derived indole, indoxyl glucuronide, suggests sequestration of tryptophan away from other constituents of the gut microbiota by *L. reuteri*. Similarly, depletion of acetylated kynurenine indicates competition with the host for tryptophan, with *L. reuteri* actively shifting the balance towards bacterial metabolites and away from the mammalian kynurenine pathway (Table S33). Further, *L. reuteri* drove a marked tryptophan-dependent increase in a variety of imidazole containing metabolites (Fig. 8D, E, G–J and Table S34). Examination of bacterial monocultures revealed that *L. reuteri* also produces a wide array of similar imidazoles in vitro, including two (imidazole propionate and 1-methyl-5-imidazoleacetate) whose production was enhanced by addition of tryptophan (Fig. 8F). Together, these data identify that *L. reuteri*-driven enhancement of autoimmunity is associated with elevation of novel tryptophan-derived imidazoles, with a reduction in indoxyl and mammalian kynurenine metabolites. .

### *L. reuteri* metabolites activate the aryl hydrocarbon receptor and augment IL-17 production by T cells in vitro

**Fig. 9:**
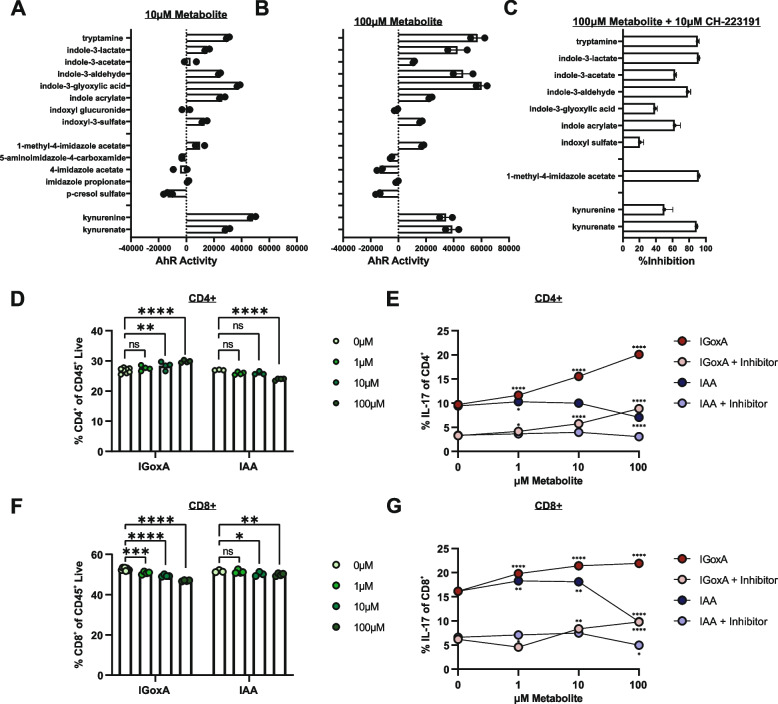
*L. reuteri* metabolites are ligands for the aryl hydrocarbon receptor and sufficient to elicit a Th17 immune response. Selected metabolites identified in *L. reuteri* monoculture or serum UPLC-MS/MS were analyzed in a cell-based luciferase assay for capacity to activate the AhR at **A** 10μM and **B** 100μM treatment of each individual metabolite. Percent inhibition of AhR activation by each metabolite was calculated between maximal luciferase response at 100μM and activity following pre-treatment for 4h with the AhR antagonist, CH-223191 (**C**). Splenocytes were differentiated under Th17 conditions with or without IGoxA, IAA, and/or AhR antagonist treatment followed by intracellular cytokine staining and flow cytometry. Major effects of metabolite treatment included frequency of CD4^+^ T cells of the total CD45^+^Live population (**D**), percent IL-17 production in CD4^+^ T cells (**E**), frequency of CD8^+^ T cells of the total CD45^+^Live population (**F**), and the percent IL-17 production in CD8^+^ T cells (**G**). Symbols indicate a significant difference between 0μM treatment and the indicated metabolite concentration as follows, *, *P* < 0.05; **, *P* < 0.01; ***, *P* < 0.001; ****, *P* < 0.0001. Results include at least (3) technical replicates per condition

To determine if metabolites identified through profiling of *L. reuteri* monocultures and the serum of *L. reuteri*-colonized mice function as ligands for AhR, we leveraged a cell-based reporter assay that links luciferase expression to ligand-dependent AhR activation. A total of 15 metabolites were selected for treatment at 1μM, 10μM, or 100μM, with and without the AhR antagonist CH-223191. All indole derivates tested activated the AhR in a concentration-dependent manner (Fig. 9A, B and Fig. S8). Interestingly, tryptamine and indole-3-glyoxylic acid, as novel *L. reuteri* tryptophan metabolites, most robustly induced AhR activation. Moreover, indole-3-glyoxylic acid was the least responsive to the AhR antagonist (Fig. 9C), suggesting high affinity binding to the receptor, enhanced diffusion across the cellular membrane, or differential sensitivity to inhibition by the AhR agonist CH-223181 [101]. Of the four *L. reuteri*-produced imidazoles, 1-methyl-4 imidazole acetate ability to function as an agonist rivaled that of indole-3-acetate (Fig. 9A, B), a *Lactobacillus*-specific metabolite previously established to activate the AhR [59]. Surprisingly, 5-aminoimidazole-4-carboxamide, 4-imidazole acetate, and p-cresol sulfate inhibited baseline AhR activity (likely activation of AhR by ligands present in the culture media) (Fig. 9A, B), suggesting complex regulation of receptor activity by *L. reuteri*-produced metabolites. Taken together, *L. reuteri* produces a wide array of tryptophan-derived metabolites with the capacity to serve as either agonists or antagonists for the immunomodulatory AhR, including high affinity binding ligands.

To determine the immunological impact of AhR activation by *L. reuteri*-produced metabolites, we examined the effect of select metabolites on Th17 polarization of lymphocytes, as assessed by intracellular cytokine staining and flow cytometry (Fig. 9D–G and Fig. S9). Indole-3-glyoxylic acid treatment prompted a modest expansion of CD4^+^ T cells at the expense of CD8^+^ T cells in culture (Fig. 9D), with a robust concentration-dependent increase in IL-17 production in both CD4^+^ and CD8^+^ T cells (Fig. 9E). By contrast, the comparatively weak AhR activator, indole-3-acetate, reduced CD4^+^ T cell numbers and IL-17 production by both CD4^+^ and CD8^+^ cell production (Fig. 9F, G). Further, IL-17 production was inhibited by treatment with the AhR antagonist, CH-223191 (Fig. 9E, G), confirming an AhR-dependent response to both metabolites. Notably, CH-223191 suppressed IL-17 production to a lower baseline independent of indole addition, most likely representing suppression of AhR activation by ligands present in the cell culture media (Fig. 9E, G), as previously documented [102]. Taken together, these data demonstrate that AhR activation by distinct indole derivatives can augment IL-17 production in a manner that is highly ligand-specific.

## Discussion

The gut microbiome has been implicated in a variety of autoimmune diseases, ranging from rheumatoid arthritis and systemic lupus erythematosus to inflammatory bowel diseases and multiple sclerosis. Despite this fact, moving away from correlative studies that characterize shifts in gut microbial community architecture towards mechanistic studies that causally link individual species to disease perturbation has remained challenging. We and others have shown that a single species of gut microbiota, *L. reuteri,* is associated with exacerbation of autoimmunity in the context of commensal colonization [86, 87, 103]. Interestingly, *L. reuteri* was also shown to ameliorate CNS autoimmunity when administered daily as a probiotic [61, 104, 105]. The need to mechanistically reconcile disparate findings such as these highlight current challenges in the study of the gut microbiome at large and suggest the need for integrative “omics” analyses incorporating both genomic and metabolomic methodology to dissect context-specific interactions between host and gut microbiome. To that end, using a multiomic approach, we have demonstrated that *L. reuteri-*dependent enhancement of autoimmunity is mechanistically driven by the availability of host dietary tryptophan, reshaping the host immune response and imparting a unique serum metabolomic signature. Moreover, through an in-depth genomic and metabolomic study of *L. reuteri*, we highlighted the nuance of interconnected bacterial and host tryptophan metabolism and identified a wide array of novel tryptophan-dependent metabolites with potential impact on EAE/MS pathogenesis.

Tryptophan metabolism in *Lactobacillus* species is thought to predominately occur through the indole pyruvate pathway, producing a wide array of immunomodulatory indole derivates. However, direct evidence for conservation of the main enzymes involved in this pathway is scant, often relying on experimental characterization within other bacterial genera [92, 96, 106, 107]. Here, we provide an extensive genomic characterization of the tryptophan pathway in *L. reuteri*, confirming the presence of ArAT as a diverse family of enzymes. Previous studies have shown that ArAT-driven indole production by *L. reuteri* is key for balancing mucosal immunity through AhR-driven IL-22 production [59]. Yet, inactivation of one of the *araT* loci does not alter direct AhR activation potential [108]. One possible explanation is the genomic redundancy of ArAT exemplified by our own data in *Lactobacilli* and as previously identified in *E. coli*, the functional consequences of which warrant further experimental exploration [92, 107]. Further, we identified three key enzymes downstream of the ArAT in the bacterial tryptophan pathways, FldH, AmiE, and possibly TrpD. The existence of the latter is supported by tryptamine production by *L. reuteri* monoculture data and genomic evidence at ~40% sequence homology to the known enzyme identified in *Clostridium sporogenes* [96]*.* Additionally, conservation of AmiE *by L. reuteri* has not been reported previously. Encoding an intermediate enzyme within the indole pathways like AmiE indicates that *L. reuteri* may work in concert with other constituents of the gut microbiome to produce immunomodulatory indoles. Together, these data highlight the inherent flaw in reliance upon annotation-based enzymatic identification, emphasize the importance of metabolomic analysis to substantiate genomic-based predictions, and despite general acceptance of conservation of these enzymatic steps in *Lactobacilli* at large, emphasize the need to formally validate these pathways. Moreover, even within a single bacterial species, one must consider genetic differences among strains [109], although our data (Fig. 2B) suggest that the presence of the major tryptophan metabolizing enzymes is conserved among most strains of *L. reuteri*.

Our data demonstrate, somewhat surprisingly, that depletion of dietary tryptophan strongly suppresses EAE severity. A single randomized controlled trial evaluated the effect of tryptophan supplementation directly in MS patient cohorts [110]. While improvement in memory was observed, the study was not designed to specifically assess disease activity (disability scores, relapse rates, MRI) [110]. However, three previous studies did characterize the role of dietary tryptophan in CNS autoimmunity in the EAE mouse model [111–113]. Lanz et al. administered tryptophan by daily gavage starting just prior to disease onset of a standard EAE course [112]. Although this was sufficient to raise blood levels of tryptophan, ultimately there was no impact on disease pathogenesis [112]. Conversely, a tryptophan-depleted diet starting on day 22 of EAE exacerbated disease, a phenotype that could be rescued by tryptophan supplementation [113]. In a similar dietary invention model to our own, Sonner et al. modulated dietary tryptophan directly in feed, providing mice with either tryptophan-deficient diet or normal chow containing ~0.3% tryptophan for 1–2 weeks prior to disease induction through end of disease course. Consistent with our own data, restriction of dietary tryptophan completely abolished EAE pathogenesis. Moreover, broad shifts in gut microbiota were observed, including a depletion of *Lactobacillus* during tryptophan restriction and EAE suppression, an observation that adds relevance to our own studies implicating *L. reuteri* tryptophan-dependent enhancement of CNS autoimmunity. Interestingly, tryptophan depletion also ameliorated disease in other models of autoimmunity including systemic lupus erythematosus and collagen-induced arthritis [114–116]. Importantly, previous studies failed to pinpoint causative gut microbial species associated with enhancement of autoimmunity in the presence of tryptophan. Consequently, divergent outcomes between tryptophan modulation in these studies may well be in part microbiota-driven as was the case between B6, B6+*L. reuteri* and PWD microbiota colonized mice in our study (Fig. 4). This is exemplified by the reduced suppressive effect of tryptophan restriction in mice colonized with PWD microbiota, perhaps owing to the presence of species that do not require tryptophan to exacerbate CNS autoimmunity. Further, the differential response to tryptophan restriction between the PWD microbiota and B6+*L. reuteri* microbiota, both contexts wherein *L. reuteri* is present, emphasizes the inherent limitation of monocolonization studies (i.e., the full microbiota context cannot be considered). Taken together, and perhaps most importantly, these data suggest that the influence of gut microbiota-derived tryptophan metabolites functions primarily in the context of disease susceptibility rather than progression.

While dietary intervention to directly manipulate tryptophan in MS patients has not been fully explored, profiling of both bacterial and host tryptophan metabolites has been quite extensive. Despite often disparate findings between cohorts, elevated levels of neuroprotective kynurenate in the cerebrospinal fluid have been observed in multiple studies during active disease, perhaps as a compensatory mechanism, with levels falling during remission [36–38, 51]. Because kynurenate cannot cross the blood-brain barrier, CNS levels depend on active transport of peripheral kynurenine. Consequently, the observed active depletion of kynurenine/acetylated kynurenine by *L. reuteri* in favor of kynurenate (Figs. 3C, K, L and 8M) may well globally lower neuroprotective levels in the CNS. While the conversion of kynurenine to kynurenate is predominantly associated with the mammalian host and was thus a surprising finding in the context of *L. reuteri* monoculture, some evidence in bacteria and yeast suggest this process may be more broadly conserved [117, 118]. Bacterial indoles, including indole-3-acetate and indole propionate are increased in secondary progressive and relapsing remitting MS, respectively [31, 33]. Consistently, in our study, EAE suppression by dietary tryptophan depletion was associated with decrease in a wide array of bacterial indoles and host-derived mammalian tryptophan metabolites (Fig. 6). However, despite producing a variety of known and novel indoles in vitro, colonization by *L. reuteri* (in the context of a normal microbiota) failed to elicit a measurable increase in serum levels of these metabolites by day 30 of EAE. Interestingly, in low-tryptophan-fed mice, *L. reuteri* was sufficient to increase serum indole propionate despite similar EAE suppression in mice replete of or lacking this species. Moreover, a variety of tryptophan-dependent metabolites that are structurally related to indoles were modulated by *L. reuteri* in the context of autoimmune exacerbation. In two recent studies, neurotoxic cresols were found to be increased in patients with MS, an observation echoed in our own data [63, 100]. In our study, both p-cresol sulfate and glucuronide were elevated in a tryptophan-dependent manner, suggesting these metabolites, as with the MS patient studies, correlate with enhanced autoimmunity (Fig. 6C). Moreover, *L. reuteri* was found to directly produce both metabolites in monoculture, with elevation of the sulfate derivate occurring in a tryptophan-dependent manner (Fig. 7F). Monoculture data was supported by *L. reuteri-* and tryptophan-dependent elevation of p-cresol sulfate in serum from naïve mice (Fig. 7E), suggesting perhaps the alteration of cresol abundance may predate disease onset, an interesting hypothesis to be tested in human populations, contributing to disease progression and/or serving as future MS predictive biomarkers. Interestingly, in one of the two aforementioned studies, imidazole derivatives were also differentially abundant between MS and control groups, though change of direction is inconsistent with our own studies [63]. Importantly, we observed a high degree of overlap between *L. reuteri* imidazole derivatives produced in monoculture and those modulated systemically in the context of high dietary tryptophan in vivo (Fig. 8E–J). Moreover, although the specific novel imidazoles examined here were not previously known to modulate the AhR, some evidence does exist to suggest either canonical AhR-dependent CYP1A or non-canonical immunomodulatory CYP1A activation by imidazoles [119, 120]. Additionally, evidence of antagonist activity by imidazole derivatives countering TCDD-induced AhR activation [121] indicates that this metabolite class can function as either activators or inhibitors of the AhR, a finding that is consistent with our own (Fig. 9A–C). Combined with recent MS patient metabolomic data, these data suggest that imidazoles represent a new class of AhR modulating metabolites produced by constituents of the gut microbiota, including *L. reuteri*, with the specific derivatives likely having divergent immunomodulatory function. Taken together, dietary tryptophan-dependent regulation of CNS autoimmunity is marked by altered abundance of a wide array of classic tryptophan-associated metabolites including indoles and kynurenines, as well as novel cresol and imidazoles in vivo, which are directly produced by *L. reuteri* in monoculture.

AhR activation by *Lactobacillus-*specific metabolites have been broadly characterized as eliciting beneficial immunological outcomes. In EAE, indoles are proposed to exert anti-inflammatory effects via AhR activation in microglia or astrocytes, correlating with the abundance of *L. reuteri* [122, 123]. *L. reuteri*-produced indole-3-aldehyde also bolsters mucosal immunity through AhR-dependent IL-22 production [59]. Importantly, in the EAE model, AhR activation can modulate CNS autoimmunity in divergent directions in a ligand-specific fashion, with FICZ exacerbating disease and TCDD and tryptamine ameliorating disease [124–126]. Interestingly, though FICZ has a higher binding affinity to the AhR than does TCDD, it can be rapidly metabolized, pointing to both strength and duration of activation as important for immunological outcome. In vitro, the AhR is required for maximal IL-17 production in T cells [102, 125, 127], with both TCDD and FICZ promoting Th17 differentiation. While indole-3-lactate reduces Th17 differentiation in vitro, correlating with a reduction in CD4^+^ IL-17 production in the spleen and spinal cord in EAE [61], activation of the AhR by kynurenine supports generation of Tregs [128]. Here, we have demonstrated a wide array of *L. reuteri-*dependent metabolites serve as either agonists or antagonists of the AhR and while the strong AhR activator, IGoxA, robustly supports maintenance of CD4^+^ T cell and IL-17 production, and the weak AhR activator, IAA, displays a suppressive effect (Fig. 9G). Further mechanistic study is needed to determine the precise molecular mechanisms leading to divergent consequences of AhR signaling. Moreover, precise experimental characterization of bacterial pathways leading to AhR ligand production is needed within the context of the complete gut microbiota, with the combined effect on AhR-driven immunomodulation explored, to fully understand the interplay between microbial and host tryptophan metabolism in AhR activity as it pertains to autoimmunity.

The gut microbiome displays remarkable inter-individual variation in healthy populations, driven in part by host genetics and various layered environmental influences. This fact likely is a major driver in the stark variation between MS patient cohort studies examining changes in the gut microbiome as associated with disease and is further confounded by the use of disease-modifying therapies. Importantly, while the abundance of specific taxa might vary greatly from person to person, the metabolic output of a healthy gut microbiome is relatively well conserved. Consequently, leveraging bacterial metabolic interplay with the host represents an attractive path forward to better understand the dysbiotic state as it pertains to disease onset, pathogenesis, and therapeutic intervention strategies. Our studies emphasize the utility of employing a multipronged approach in defined microbiota systems to tease apart complex bacterial-host metabolic pathways and mechanistically link them to disease pathogenesis. Through this approach, for the first time, we were able to demonstrate that tryptophan-dependent EAE pathogenesis can be causally linked to a keystone gut commensal species, *L. reuteri*. Future efforts, employing further reductionist methods will be increasingly important to interrogate complex networks of metabolic cross-talk between constituents of the gut microbiome and the host.

## Conclusions

In summary, we find that metabolism of dietary tryptophan by specific gut commensal microbes such as *L. reuteri* can unexpectedly promote experimental autoimmune disease. Analysis of the genome and metabolome of *L. reuteri* reveal a diverse metabolic arsenal for tryptophan catabolism, and restriction of tryptophan bioavailability ameliorates EAE, reduces the abundance of indole derivatives, and modulates CNS-targeted T cell responses. Together with emerging studies in MS patients demonstrating dysregulated bacterial tryptophan metabolism, our studies confirm a causal role for this metabolic axis and provide a mechanistic basis through the identification of a causative species, its genetic pathways, and potential downstream metabolites.

## Methods

### *Lactobacillus* species isolation

A total of 19 PWD/PhJ (PWD) mice (9 males and 10 females) were previously screened by 16S sequencing and found to be positive for *L. reuteri* [87]. All subsequent PWD mice screened by qPCR with species-specific primers also were positive for *L. reuteri. L. reuteri* was isolated previously [87] as follows. Total cecal contents from three male wild-derived inbred (PWD) strain mice were harvested anaerobically, pooled, and resuspended in MRS medium (Thermo Fisher, Inc, USA) supplemented with 0.25g/L L-cysteine and 20μg/ml vancomycin and adjusted to pH 5. Contents were incubated anaerobically overnight at 37°C, at 200 rpm, and resulting cultures were streaked for isolation onto agar medium of the same formulation. Single colonies were selected based on morphology consistent with lactic acid bacteria, cultured overnight in 5 ml MRS medium of the same formulation and cryopreserved, followed by DNA extraction through standard boiling preparation, and screening by qPCR using species-specific primers. Positive clones were recovered from glycerol stocks, cultured in vancomycin-free medium to confirm purity with repeat qPCR analysis. *L. murinus* and *L. johnsonii* were isolated in the same manner from three male classic inbred C57BL/6J (B6) mouse strain cecal or stomach contents respectively, with the exclusion of vancomycin in the selection of *L. johnsonii*. B6 and PWD mice were fed standard chow at 0.28% tryptophan (PROLAB RMH 3000 cat# 5P00) prior to isolation of *Lactobacilli.* For colonization studies, three isolates per species were grown to log-phase, adjusted to OD600=0.5 with fresh culture medium, mixed at equal volumes, and cryopreserved, followed by repeat qPCR validation of each pooled stock. All bacterial isolates are readily available from the authors upon request.

### Whole genome sequencing and assembly

DNA was extracted from two pure *Lactobacillus* isolates per species as cryopreserved above using DNeasy UltraClean Microbial Kit (Qiagen, USA). Concentration and quality were assessed using Qubit (250 ng cut-off ranging 160–268 ng/μl) and 2100 Bioanalyzer High Sensitivity DNA Analysis (predominant peak at upper marker size 10,390bp) respectively. Following fragmentation free library preparation and barcoding (Ligation Sequencing Kit, SQK-LSK10 and Native Barcoding Expansion, EXP-NBD104, Nanopore, USA), isolates were multiplexed and sequenced on a Nanopore GridIon X5 Long Read Sequencer (Flow Cell ID, FAL58627, Nanopore, USA) with basecalling using Guppy version 3.2.8. Sequence quality was assessed using NanoQC and Nanoplot within NanoPack version 1.0.1, filtered at a quality score of 7 or greater based on Oxford Nanopore recommendations for long-read sequencing, trimmed using Trimmomatic version 0.39 (Leading:10, Trailing: 10, and Headcrop: 50), and bacterial genomes were assembled using Flye version 2.6 and Unicycler version 0.4.8. Draft assemblies were compared using the Quality Assessment Tool for Genome Assemblies (QUAST) version 5.0.2 [129]. Flye assemblies were selected for further annotation and analysis based on overall quality assessment (N50, misassembled and unaligned contigs or contig bases, genes and operons covered, etc.). Assemblies were annotated using PROKKA version 1.14.5 and the Pathosystems Resource Integration Center (PATRIC). Draft genomes are publicly on PATRIC (ID: 186826.38 *L. reuteri* Isolate 1, genome ID: 186826.48, *L. reuteri* Isolate 2, genome ID: 186826.44*L. murinus* Isolate 1, genome ID: 186826.45*L. murinus* Isolate 2, genome ID: 186826.46 *L. johnsonii* Isolate 1, and genome ID: 186826.47 *L. johnsonii* Isolate 2) and the Integrated Microbial Genomes and Microbiomes (IMG/M) (ID: 2870538698 *L. reuteri* Isolate 1, ID: 2870542885 *L. reuteri Isolate* 2, ID: 2870555403 *L. murinus* Isolate 1, ID: 2870560610 *L. murinus* Isolate 2, ID: 2870565740 *L. johnsonii* Isolate 1, and ID: 2870569636 *L. johnsonii* Isolate 2).

Assemblies were queried against the NCBI non-redundant prokaryotic genomes database internal to the Microbial Genomes Atlas Online (MIGA) (Database update 12/28/2019) [89]. Taxonomic classification was inferred by the maximum average amino acid identity (AAI) against all genomes in the database with *p*-values estimated from the distribution in all the reference genomes in NCBI’s RefSeq at each taxonomic level as a readout of classification probability. Average nucleotide identity (ANI) and AAI tables of maximally the top-50 reference hits in the database were extracted and graphed as *x-y* scatter plots to determine nearest subspecies phylogenetic neighbors. To generate phylogenic trees of isolates and MIGA-identified nearest phylogenetic neighbors, the interfered proteome from PROKKA isolate annotations or as publicly available in NCBI for applicable reference genomes were uploaded to the ANI/AAI-Matrix calculator [90]. The resulting phylogenetic tree based on AAI was edited using the interactive tree of life (iTOL) [130]. Host origin for each strain/species was manually curated. Core and accessory genomic elements were differentiated and mapped to the KEGG database using the Bacterial Pan Genome Analysis (BPGA) pipeline version 1.3.0 using PROKKA annotated proteomes of each isolate. Core, accessory, and unique genomic KEGG orthology identifiers were extracted for KEGG enrichment analysis using clusterProfiler version 3.10.1 [82]. Abundance profiles of the top-20 COG elements were identified using the compare genomes tool at the Integrated Microbial Genomes & Microbiomes (IMG/M) system at the Joint Genome Institute (JGI) [131].

Bacterial tryptophan-associated enzymes were identified based on previous studies [53, 132–134] and are detailed in supplemental (Table S8), including TNA, TMO, TrpD, ArAT, ALD, IPDC, FldH, and AmiE. Briefly, enzyme commission numbers where available or alternatively enzyme names were queried in PATRIC, PATRIC Global Family (PGF) cross-genus identifiers were extracted and compiled for query within isolate genomes. Protein sequences of isolate identified enzymes were analyzed using InterProScan for additional functional prediction, and sequence homology to the previously experimentally validated enzymes within other bacterial species was determined using Blastp at 30% cross-genus identify and 90% identity within each species.

### Bacterial culture

A pooled gavage stock of three pure *L. reuteri* isolates described in the “*Lactobacillus* species isolation” section was grown anaerobically in brain heart infusion (BHI) medium (Sigma, USA) supplemented with 5 g/L yeast extract (Sigma, USA), 0.5 g/L-cysteine (Thermo Fisher, USA), 0.2 ml Vitamin K (Sigma, USA), 0.2 mg/L hemin (Sigma, USA) and 5% each of newborn calf serum, (Thermo Fisher, USA), horse serum (Thermo Fisher, 26050088), and sheep serum (Millipore Sigma, USA). Tryptophan (Thermo Fisher, USA) was supplemented into basal medium at 1 mM as applicable. Bacterial cultures were grown for 24 h at 37°C without shaking and centrifuged at 3500 rpm for 10 min, the supernatant was filtered at 0.22 μm, and aliquots were stored at −80°C until analysis via ultrahigh-performance liquid chromatography-tandem mass spectroscopy (UPLC-MS/MS) (Metabolon Inc. Durham, NC) [97]. Medium only bacteria-free cultures (with and without 1 mM supplementation) were processed and analyzed in tandem serving as a control.

### Bacterial RNA extraction and aromatic aminotransferase mRNA expression quantification

*L. reuteri* was cultured as described in the bacterial culture section for 4 or 24 h. Cultures were centrifuged at 8000 rpm for 10 min, the supernatant was removed, and pellets were then resuspended in 800 μl TRIzol reagent (Invitrogen, USA) pre-warmed to 55°C and incubated at 55°C for 10 min. TRIzol suspended bacteria was transferred to ZR BashingBead Lysis Tubes (Zymo Research, USA), homogenized for 30 s using a Mini-Beadbeater (Biospec Products, USA), and RNA was extracted using a Direct-zol™ RNA Miniprep kit (Zymo Research, USA). RNA concentration was determined by nanodrop (Thermo Scientific NanoDrop 2000 Spectrophotometer), and cDNA was synthesized using the qScript cDNA Super MIX kit (QuantBio, USA) according to the manufacturer’s instructions. *araT* expression was quantified by qPCR with the DyNAmo ColorFlash SYBR Green kit (Thermo Fisher Scientific, USA), using *araT*-specific primers performed on a Quant Studio 3 Real-Time PCR System (Thermo Fisher Scientific, USA). Data was normalized to a pan-eubacteria-specific primer set [87] with relative abundance calculated by a comparative Ct method formula 2^−(deltaCt)^. Primer sets are available in Table S35.

### Microbial DNA isolation and *Lactobacillus* quantification

Fecal samples were collected by placing individual mice into empty cages without bedding and waiting for them to defecate followed by brief storage on ice and long-term storage at −80°C until extraction. DNA was extracted from fecal pellets using the QIAamp PowerFecal Pro DNA extraction kit (Qiagen, USA) with DNA quality and quantity assessed by NanoDrop. Bacterial abundance was quantified using species-specific primers against the 16S rRNA gene by qPCR and normalized to a pan-eubacteria specific primer set as described above. Primer sets are listed in Table S35.

### Animals and microbiota transplantation

Gut microbiota transplantation (GMT) was performed as previously described [87]. Briefly, cecal contents were cryopreserved from B6 or PWD mice under anaerobic conditions, flash frozen at a final concentration of 20% glycerol in Hungate tubes, and stored at −80°C until use. Germ-free (GF) 4–5-week-old C57BL/6J mice were purchased from the National Gnotobiotic Rodent Resource Center at University of North Carolina School of Medicine (Chapel Hill, NC, USA), shipped in sterile crates that were opened under a laminar flow hood and immediately inoculated by gastric gavage with 100μl of cryopreserved PWD or B6 cecal content. To generate the B6+*L. reuteri* microbiota, GF mice received 100μl of the B6 microbiota supplemented with 100μl *L. reuteri* at 10^9^ CFU. The resulting ex-GF mice, served as breeding pairs for a vertical gut microbiota transmission model. All animals were maintained under barrier conditions with sterilized food, water, and caging, with handling minimized to ensure minimal introduction of additional microbes or cross-contamination. The experimental procedures used in this study were approved by the Animal Care and Use Committee of the University of Vermont.

### Animal husbandry practices related to preservation of experimental microbiota integrity

To maintain the integrity of experimental microbiota murine cohorts, strict handling orders and the use of aseptic technique was instituted to avoid cross-contamination. All animal husbandry including cage changes were managed by trained lab personnel as follows: Peroxigard™ is sprayed liberally to clean a biosafety cabinet, the hood wiped free of debris and then sprayed again, and allowed to dry for a minimum of 10 min prior to the start of work and between all experimental groupings. Fresh gloves and gown are also used for each experimental microbiota grouping. Autoclaved cages, irradiated vacuum-sealed food, and filter-sterilized water are used to make fresh cages within the biosafety cabinet in bulk to avoid exposure to animals with individual prepared cages sprayed into the hood as needed thereafter. Handling order for this study was as follows: PWD (naturally contains *L. reuteri*) breeding pairs, PWD experimental cages, B6 (naturally contains *L. murinus*) breeding pairs, B6 experimental cages, B6+*L. reuteri* breeding pairs, B6+*L. reuteri* experimental cages. Experimental diets were portioned to autoclaved containers prior to the start of experiments such that each microbiota group had its own stock container. Dietary groupings were treated as separate microbiota cohorts with the following handling order: PWD 0.02% Trp diet, PWD 0.8% diet, B6 0.02% Trp diet, B6 0.8% Trp diet, B6+*L.reuteri* 0.02% Trp diet, and B6+*L.reuteri* 0.8% Trp high diet.

### Induction and evaluation of EAE

EAE was induced in ex-GF-B6 GMT recipients with the 2×MOG35-55/CFA protocol [135]. Briefly, mice were injected subcutaneously on day 0 (lower flank) and day 7 (upper flank) with 50μl per flank of 0.1mg of myelin oligodendrocyte glycoprotein peptide 35-55 (MOG35-55) (New England Peptide, Inc. MA, USA) emulsified in PBS and 50% complete Freund’s adjuvant (CFA; Sigma, USA) supplemented with an additional 4 mg/ml Mycobacterium tuberculosis H37Ra (Difco, USA). Starting on day 10, mice were scored, as follows: 1—loss of tail tone, 2—loss of tail tone and weakened hind limbs, 3—hind limb paralysis, 4—hind limb paralysis and incontinence, 5—quadriplegia or death. Significant differences in disease course were determined using Friedman’s non-parametric two-way ANOVA, as previously described [135], using the treatment×time interaction term to evaluate differences. Overall disease severity was calculated using the area under the curve (AUC) of each respective EAE course and graphing total peak area and standard error of the mean, with significance calculated by one-way ANOVA and Šidák correction for multiple comparisons.

For dietary intervention studies, defined microbiota colonized mice were randomized to a low 0.02 or 0.8% high-tryptophan diet (TD.200350 and TD.200352, Envigo Teklad Diets, Madison, WI) and housed at 2–3 mice per cage 1 week prior to EAE induction at 8–12 weeks of age. Diets were vacuum packaged, irradiated, stored at 4°C until use, refreshed weekly in experimental cages, and provided on the floor of the cage to mice reaching a clinical score of 3 or greater (when hind limb paralysis was noted) along with Napa nectar. Weights were taken every other day for the duration of the experiment. Prior to randomization to high- and low-tryptophan diets, defined microbiota colonized mice were fed a 0.30% tryptophan-containing diet (Prolab Isopro RMH 3000 cat# 5P75).

### Flow cytometry

To characterize CNS-infiltrating cells at day 30 post-EAE induction, mice were euthanized by exsanguination by transcardial perfusion with PBS under isoflurane anesthetization. Lymphocytes were isolated from the spinal cord by Dounce homogenization to generate a single-cell suspension that was filtered with a 70-μm strainer followed by Percoll gradient (37%/70%) centrifugation and collection of the interphase. For intracellular cytokine analysis, cells were stimulated with 20 ng/ml PMA, 1 μg/ml of ionomycin and 1 mg/ml brefeldin A (Golgi Plug reagent, BD Bioscience) for 4 h. Cells were stained with the UV-Blue Live/Dead fixable stain (Thermo Fisher, USA) followed by surface staining with antibodies against CD45, CD11b, CD19, TCRβ, CD4, CD8, and TCRγδ (Biolegend, USA). For intracellular cytokine staining, cells were fixed, permeabilized with 0.05% saponin, and labeled with anti-IL-17A, anti-IFNγ, and anti-GM-CSF antibodies (Biolegend, USA). Cells were analyzed using a Cytek Aurora (Cytek Biosciences, USA). Spectral unmixing was performed with appropriate single-color controls using autofluorescence correction from an unstained group control. Data were analyzed using FlowJo software, version 10.8.1 (Tree Star Inc, Ashland, OR).

### Metabolomics

Serum was collected from mice colonized with the B6 or B6+*L. reuteri* microbiota fed a low (0.02%) or high (0.8%) tryptophan diet for 1 week (naïve mice) or 5 weeks (following a full 30-day EAE course) as outlined in Fig. 4A and B. Specifically, blood was collected by cardiac puncture, transferred to a 1.5-mL tube, allowed to clot at room temperature for 30 min, and then refrigerated 30 min. The sample was then spun down at 5000 rpm at 4°C for 5 min followed by removal of the serum from the top of the clot, transfer to a fresh 1.5-ml tube for centrifugation at 7000 rpm at 4°C for 5 min with transfer of the final serum sample to a fresh 1.5-ml tube. Samples were stored at −80°C, shipped on dry ice to Metabolon Inc. Durham, NC, for processing and analysis via UPLC-MS/MS as previously described [97]. Briefly, samples were prepared using the automated MicroLab STAR system (Hamilton Company, Franklin, MA). Proteins were precipitated by shaking for 2 min with methanol (Glen Mills GenoGrinder 2000) and centrifugation. The resulting extract was divided into five fractions, placed on a TurboVap (Zymark) to remove the organic solvent, stored overnight under nitrogen, dried, and reconstituted as follows: (1) acidic positive ion conditions optimized for hydrophilic compounds wherein the extract was gradient eluted from a C18 column (Waters UPLC BEH C18-2.1x100 mm, 1.7 μm) using water and methanol, containing 0.05% perfluoropentanoic acid (PFPA) and 0.1% formic acid (FA), (2) acidic positive ion conditions optimized for hydrophobic compounds wherein the extract was gradient eluted from the same C18 column using methanol, acetonitrile, water, 0.05% PFPA, and 0.01% FA and was operated at an overall higher organic content, (3) basic negative ion optimized conditions using a separate dedicated C18 column wherein extracts were gradient eluted from the column using methanol and water with 6.5mM ammonium bicarbonate at pH8, and (4) negative ionization following elution from a HILIC column (Waters UPLC BEH Amide 2.1x150 mm, 1.7 μm) using a gradient consisting of water and acetonitrile with 10mM ammonium formate, pH 10.8. All methods used a Waters ACQUITY ultra-performance liquid chromatography (UPLC) and a Q-Exactive high resolution/accurate mass spectrometer interfaced with a heated electrospray ionization (HESI-II) source and Orbitrap mass analyzer operated at 35,000 mass resolution. Analysis alternated between MS and data-dependent MSn scans using dynamic exclusion and ranging between 70 and 1000 m/z. Raw data was extracted, peak-identified, and QC processed using Metabolon’s hardware and software. Compounds were identified by comparison to a library of 3300 authenticated purified standards and additional mass spectral entries for recurrent structurally unnamed biochemical entities.

Data were analyzed using MetaboAnalyst, version 5.0 [136]. Data were log-transformed and mean centered, and entries with missing values for any sample were excluded. Differentially abundant metabolites were identified using one-way ANOVA or *T*-test as appropriate at a threshold of *p*≤0.05 and |FoldChange|>1.5 as consistent with the fold-change of tryptophan between low and high tryptophan conditions. Post hoc analyses used the fisher least significant difference (LSD) method. All heatmaps reflect normalized autoscaled data clustered by Euclidean distance and Ward’s linkage. Multivariate dimensionality reduction plots utilize partial least squares-discriminant analysis (PLS-DA). A manually curated list of tryptophan-associated metabolites was used to subset data for direct analysis. Raw data and data tables consistent with each analysis are included in Tables S20-S34.

### AhR activity assay and T cell in vitro culture metabolite treatment

HEK293T cells were grown in RPMI-1640 (Thermo Fisher, USA) supplemented with 10% heat inactivated one shot fetal bovine serum (Thermo Fisher, USA), 1% penicillin-streptomycin (Thermo Fisher, USA), 50 μM 2-mercaptoethanol (Thermo Fisher, USA), 2.5g/L D-glucose (Sigma, USA), 2mM L-glutamine (Thermo Fisher, USA), 10μg/ml folate (Sigma, USA), and 1mM pyruvate (Thermo Fisher, USA). Cells were plated at 2×10^4^ cells per well in 96-well plates 24 h before transfection using Lipofectamine 2000 (Thermo Fisher, USA) with pAhR and the reporter construct pGud-Luc (gift of Dr. Francisco Quintana, Harvard Medical School). Twenty-four hours later, cells were treated with individual metabolites at 100μM, 10 μM, 1μM, or 0.1μM. In some experiments, cells were pretreated for 4 h with the AhR inhibitor CH223191 (Sigma, USA) prior to individual metabolite treatment at 100μM, 10 μM, 1μM, or 0.1μM. Luciferase activity was measured 48 h later using the Pierce Firefly Luciferase Glow Assay Kit (Thermo Fisher, USA) at 613nm on a luminometer. Data was normalized to an appropriate DMSO vehicle control when assessing AhR activation. Percent inhibition was calculated between maximal luciferase response at 100 μM and activity following pre-treatment using the AhR antagonist, CH-223191.

For characterization of T cell differentiation in response to metabolite treatment, splenocytes were collected from 8–10-week-old mice and stimulated with plate-bound anti-CD3 (coated at 5 μg/ml) under Th0 (no additional cytokines) or Th17 (IL-1β, IL-23 TGFβ at 10ng/ml and IL-6 at 20ng/ml) polarizing conditions. Following 3 days of culture, cells were stimulated with 20ng/ml PMA, 1 μg/ml of ionomycin and 1mg/ml brefeldin A (Golgi Plug reagent, BD Bioscience) for 4 h, stained as described for the spinal cord samples described above, and evaluated by flow cytometry.

## Supplementary Information

**Additional file 1: Figure S1.** Full radial phylogenetic trees of *Lactobacillus* isolates and nearest subspecies neighbors. Extended taxonomic data comparing duplicate isolate draft genomes for *L. reuteri* (A-B), *L. murinus* (C-D) and *L. johnsonii* (E-F). Nearest phylogenetic neighbors, including subspecies neighbors, of each *Lactobacillus* draft genome determined by average amino acid identify (AAI) percent shared genomic content are represented as phylogenetic trees. Color gradients denote percent conserved average nucleotide identity (ANI) between each isolate and respective nearest subspecies phylogenetic neighbors.

**Additional file 2: Figure S2.** Expression levels of putative aromatic amino acid aminotransferase (ArAT) loci identified in the *L. reuteri* genome. (A) Pathway schematic of bacterial and abbreviated mammalian tryptophan metabolism from Fig. 2A. Enzymes with genomic evidence in *Lactobacillus* isolates are boxed in orange (ArAT), blue (FldH) and yellow (AmiE). (B) Heatmap of bacterial tryptophan specific enzymes with genomic evidence in *Lactobacillus* isolates. Enzymes are listed along the left in corresponding colors to the pathway in (A) with isolates and representative strains of the same species along the top and warmer colors indicating increasing copy number. (C) Expression level of *araT* loci in *L. reuteri* following 4 or 24hrs (D) of monoculture with 0 or 1mM tryptophan supplementation in brain heart infusion (BHI) medium as measured by qRT-PCR. Data is organized top to bottom corresponding to the heatmap in (B). Cultures were performed in triplicate and expression levels are normalized to a pan-Eubacterial primer set against the 16S rRNA gene. Primer sets are available in Table S35.

**Additional file 3: Figure S3.** Representative locus housing D-lactate dehydrogenase (*fldH*) and the adjacent aromatic amino acid aminotransferase (*araT*) in *L. reuteri* isolates and reference taxa. Each *araT* (red) and *fldH* (green) locus in all *Lactobacillus* isolates were compared to representative references genomes using the compare region viewer in PATRIC. The *L. reuteri* locus wherein *araT* and *fldH* are structured as a pseudo-operon that is conserved in closely related reference taxa is depicted as a schematic.

**Additional file 4: Figure S4.** Tryptophan availability rewires *L. reuteri* metabolic output in monoculture. (A) Partial least-squares discriminate analysis (PLS-DA) and (B) hierarchical clustering by Euclidean distance using Ward’s linkage represented as a heatmap of total metabolites from basal BHI medium and *L. reuteri* monocultures with or without 1mM tryptophan supplementation. Heatmap reflects top 25 differentially abundant metabolites between *L. reuteri* monocultures with or without 1mM tryptophan and analyzed by t-test at a threshold of p≤0.05 to generate a subset of metabolites most influenced by *L. reuteri* tryptophan metabolism. The resulting list was analyzed in all four experimental groups (including media alone controls) by one-way ANOVA at p≤0.05 and represented as a heatmap.

**Additional file 5: Figure S5.** Abundance dynamics of *L. reuteri* in experimental breeding pairs and within dietary intervention studies. Founder G_0_ B6-GF mice were inoculated with cryopreserved donor cecal contents from PWD, B6, or B6 cecal contents supplemented with 10^9^ CFU of *L. reuteri* and breeding pairs were established for vertical transmission to G_1_ offspring. Fecal samples were collected at 4-wks post-inoculation and in experimental offspring prior to dietary intervention (pre-diet), following 1-week of randomized diets (naive) and following a full 30-day disease course (post-EAE). Abundance of *L. reuteri* and *L. murinus* was determined by qPCR using species specific primers in (A) G_0_ breeders and G_1_ offspring and (B) throughout the course of dietary intervention. Relative abundance of *L. reuteri* at day 30 post EAE induction was correlated with disease severity as measured by cumulative disease score (CDS) using linear regression with the P value indicating significant deviation from a non-zero slop (i.e. significant correlation) for pre-diet (C), naive (D), and post-EAE (E) samples.

**Additional file 6: Figure S6.** Microbiome and dietary signatures in pooled analysis of serum metabolomic data. Serum was collected from mice fed a low or high tryptophan diet colonized with either the B6 or B6+*L. reuteri* microbiome following a 30-day EAE course and analyzed via UPLC-MS/MS as outlined in (Fig. 4A and B). Data were analyzed for all four experimental groups: B6 or B6+*L. reuteri* colonized mice randomized to a low 0.02% or 0.8% high tryptophan diet. (A) Partial least squares-discriminant analysis (PLS-DA) of total metabolites. (B) Top 10 metabolites as variables of importance in the PLS-DA projection (VIP) along component 1 responsible for segregating samples by diet. (C) Top 10 metabolites as variables of importance in the PLS-DA projection (VIP) along component 2 responsible for segregating samples by microbiome. Statistical analysis is provided in Table S24.

**Additional file 7: Figure S7.** The abundance of p-cresol sulfate and p-cresol glucuronide correlate with disease severity. Serum was collected from mice fed a low or high tryptophan diet colonized with either the B6 or B6+*L. reuteri* microbiome following a 30-day EAE course and analyzed via UPLC-MS/MS as outlined in (Fig. 4A and B). Data were analyzed for all four experimental groups: B6 or B6+*L. reuteri* colonized mice randomized to a low 0.02% or 0.8% high tryptophan diet. (A) Top 25 metabolites correlating with disease severity as measured by cumulative disease score (CDS), the sum of all daily scores over a 30-day disease course. (B) X-Y scatter plot of p-cresol sulfate and p-cresol glucuronide abundance and CDS using linear regression, with the P value indicating significant deviation from a non-zero slope (i.e. significant correlation). A zero value was entered for p-cresol glucuronide abundance in low-tryptophan-fed mice when below the limit of detection. Statistical analysis is provided in Table S31.

**Additional file 8: Figure S8*. ****L. reuteri* metabolites are ligands for the aryl hydrocarbon receptor. Selected metabolites identified in *L. reuteri* monoculture or serum by UPLC-MS/MS were analyzed in a cell-based luciferase assay for capacity to activate or inhibit the AhR at 100μM, 10μM, 1μM, and 0.1μM with or without 4hr pre-treatment with the AhR antagonist, CH223191 at 10μM. Transfection and DMSO vehicle controls are included for comparison.

**Additional file 9: Figure S9.** L. reuteri metabolites are sufficient to elicit a Th17 immune response. Splenocytes were differentiated under Th17 conditions without or without IGoxA, IAA, and/or AhR antagonist treatment at 0, 1, 10 or 100 μM followed by intracellular cytokine staining and flow cytometry. CD4^+^ and CD8^+^ T cell (A) frequency of total CD45^+^ T cells and IL-17 production (B and C) as frequency of each parent population in response to IGoxA treatment with and without AhR inhibitor. CD4^+^ and CD8^+^ T cell (D) frequency of total CD45^+^ T cells and IL-17 production (E and F) as frequency of each parent population in response to IAA treatment with and without AhR inhibitor.

**Additional file 10: Tables S1-S36.** Genomic and metabolomic raw data and analyses, metadata and primer sets used in this study.

## Data Availability

Genomic and/or metabolomic datasets generated or analyzed during this study are included in this published article and its supplementary information files. Additional datasets used and/or analyzed during the current study are available from the corresponding author on upon request.
