# Supplementary figures and images for: Lactobacillus reuteri tryptophan metabolism promotes host susceptibility to CNS autoimmunity
Source: Microbiome. 2022 Nov 23;10:198. doi: 10.1186/s40168-022-01408-7 (PMC9685921; doi:10.1186/s40168-022-01408-7)

Figure S1

A

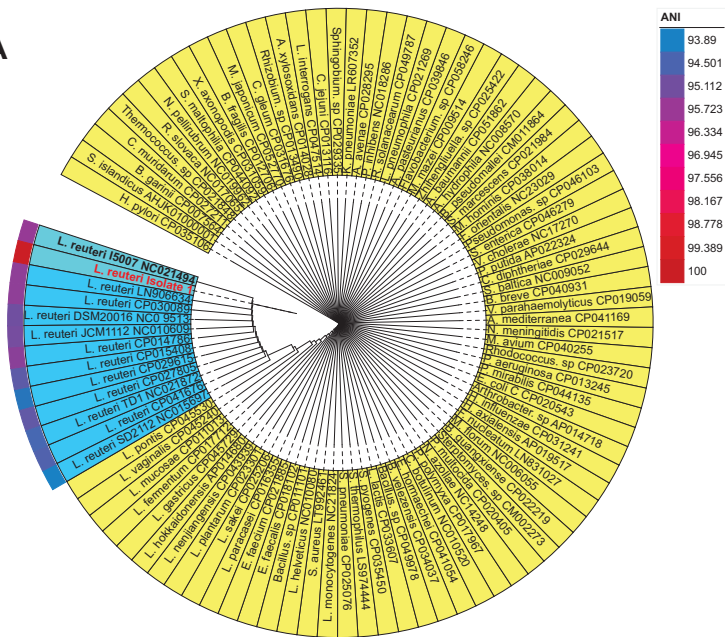

B

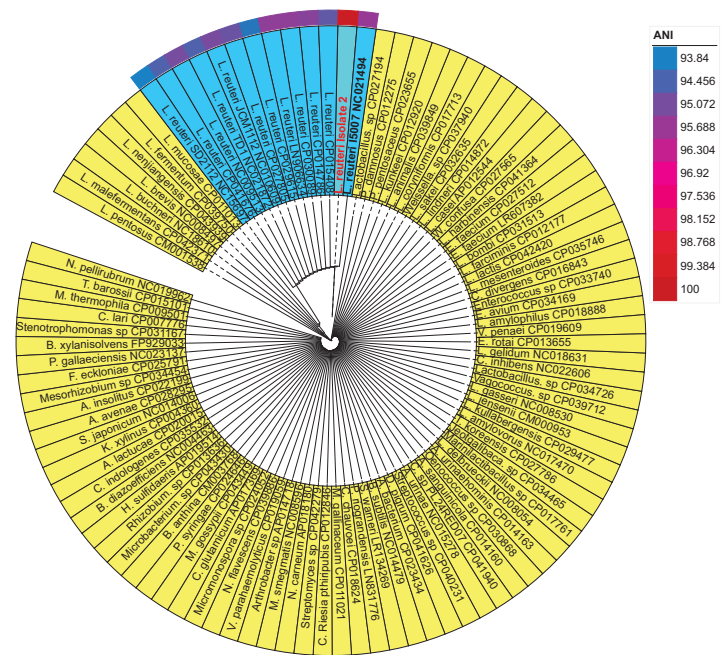

C

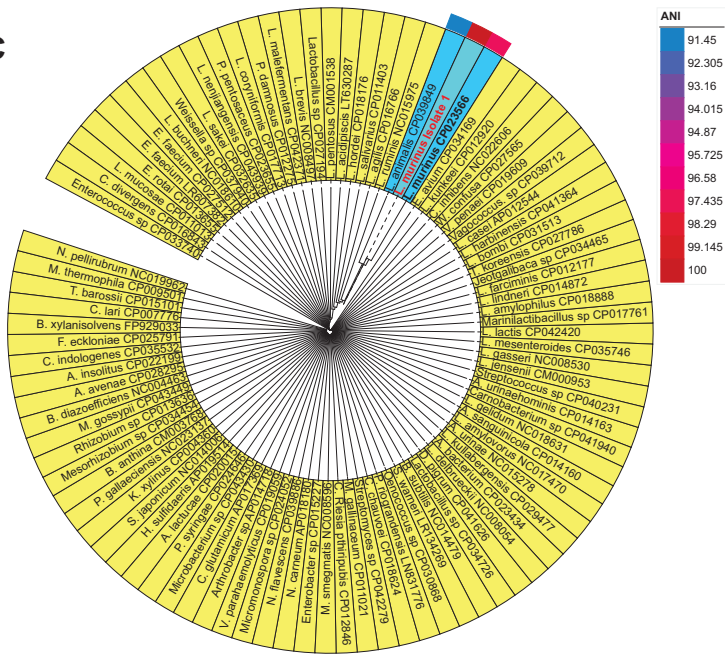

D

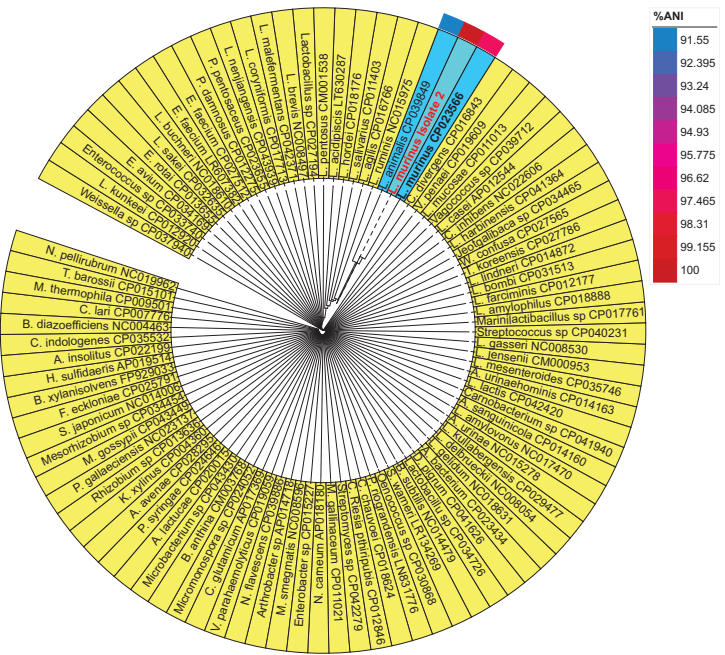

E

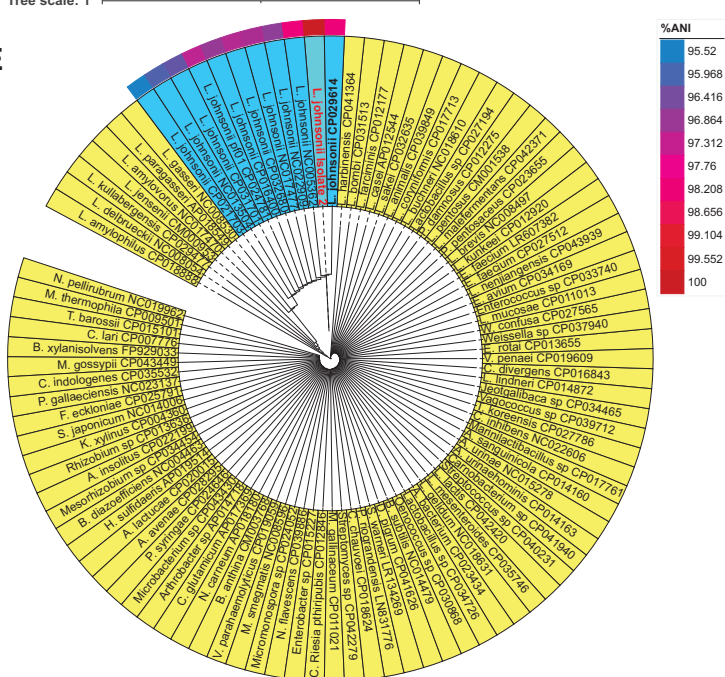

F

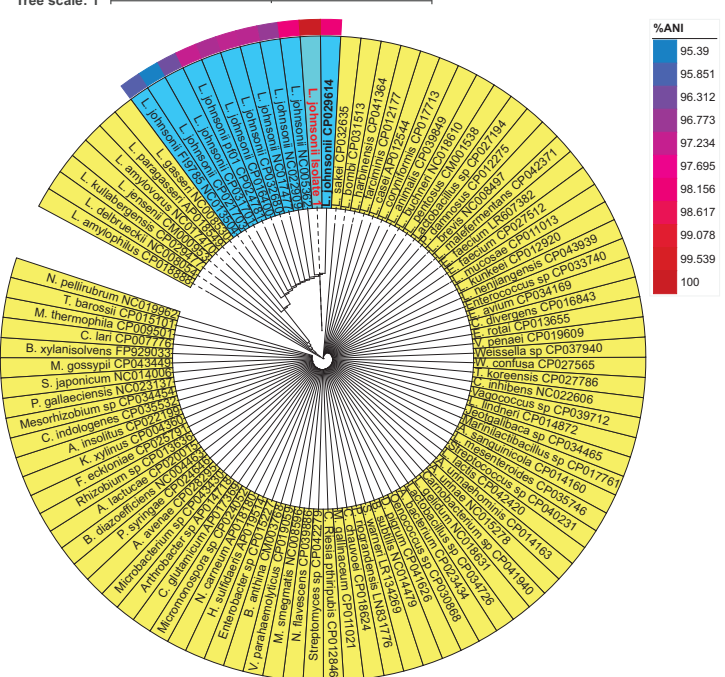

Supplement: Supplementary file 2 — Additional file 1: Figure S1. Full radial phylogenetic trees of Lactobacillus isolates and nearest subspecies neighbors. Extended taxonomic data comparing duplicate isolate draft genomes for L. reuteri (A-B), L. murinus (C-D) and L. johnsonii (E-F). Nearest phylogenetic neighbors, including subspecies neighbors, of each Lactobacillus draft genome determined by average amino acid identify (AAI) percent shared genomic content are represented as phylogenetic trees. Color gradients denote percent conserved average nucleotide identity (ANI) between each isolate and respective nearest subspecies phylogenetic neighbors. [file 40168_2022_1408_MOESM1_ESM.pdf]

**A**

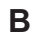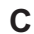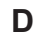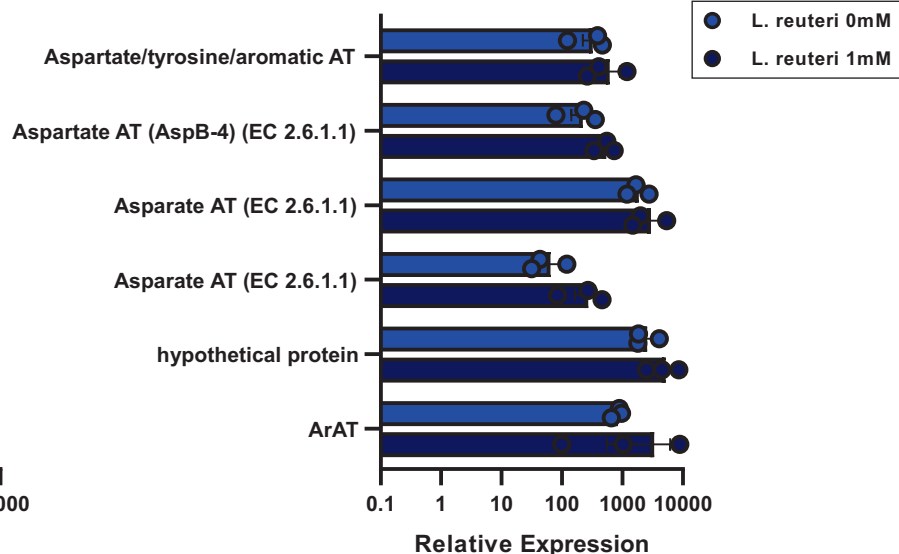

Supplement: Supplementary file 3 — Additional file 2: Figure S2. Expression levels of putative aromatic amino acid aminotransferase (ArAT) loci identified in the L. reuteri genome. (A) Pathway schematic of bacterial and abbreviated mammalian tryptophan metabolism from Fig. 2A. Enzymes with genomic evidence in Lactobacillus isolates are boxed in orange (ArAT), blue (FldH) and yellow (AmiE). (B) Heatmap of bacterial tryptophan specific enzymes with genomic evidence in Lactobacillus isolates. Enzymes are listed along the left in corresponding colors to the pathway in (A) with isolates and representative strains of the same species along the top and warmer colors indicating increasing copy number. (C) Expression level of araT loci in L. reuteri following 4 or 24hrs (D) of monoculture with 0 or 1mM tryptophan supplementation in brain heart infusion (BHI) medium as measured by qRT-PCR. Data is organized top to bottom corresponding to the heatmap in (B). Cultures were performed in triplicate and expression levels are normalized to a pan-Eubacterial primer set against the 16S rRNA gene. Primer sets are available in Table S35. [file 40168_2022_1408_MOESM2_ESM.pdf]

Figure S3

A

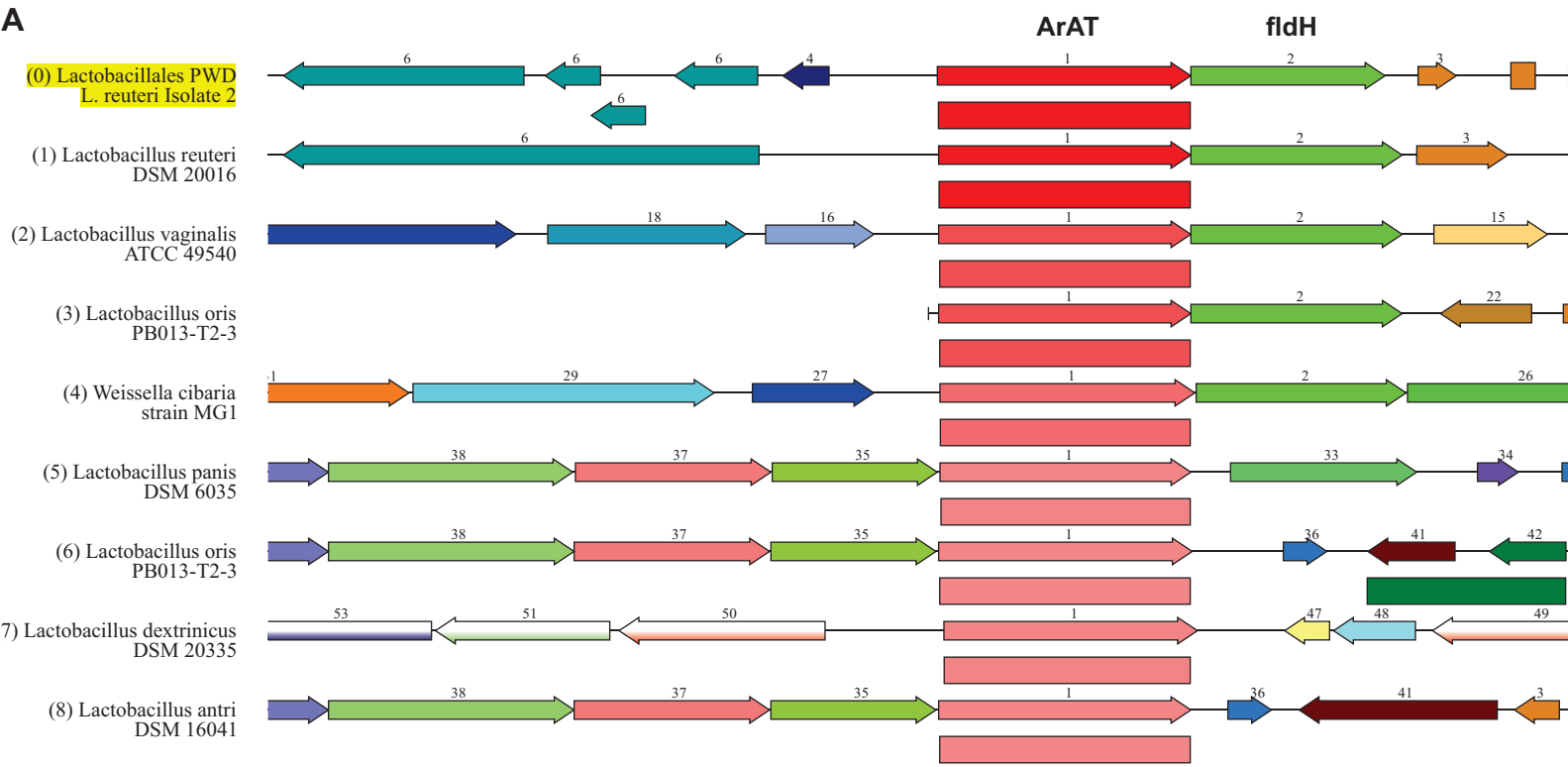

Supplement: Supplementary file 4 — Additional file 3: Figure S3. Representative locus housing D-lactate dehydrogenase (fldH) and the adjacent aromatic amino acid aminotransferase (araT) in L. reuteri isolates and reference taxa. Each araT (red) and fldH (green) locus in all Lactobacillus isolates were compared to representative references genomes using the compare region viewer in PATRIC. The L. reuteri locus wherein araT and fldH are structured as a pseudo-operon that is conserved in closely related reference taxa is depicted as a schematic. [file 40168_2022_1408_MOESM3_ESM.pdf]

Figure S4

A

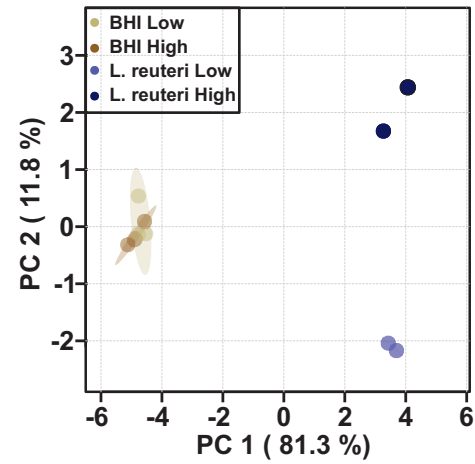

B

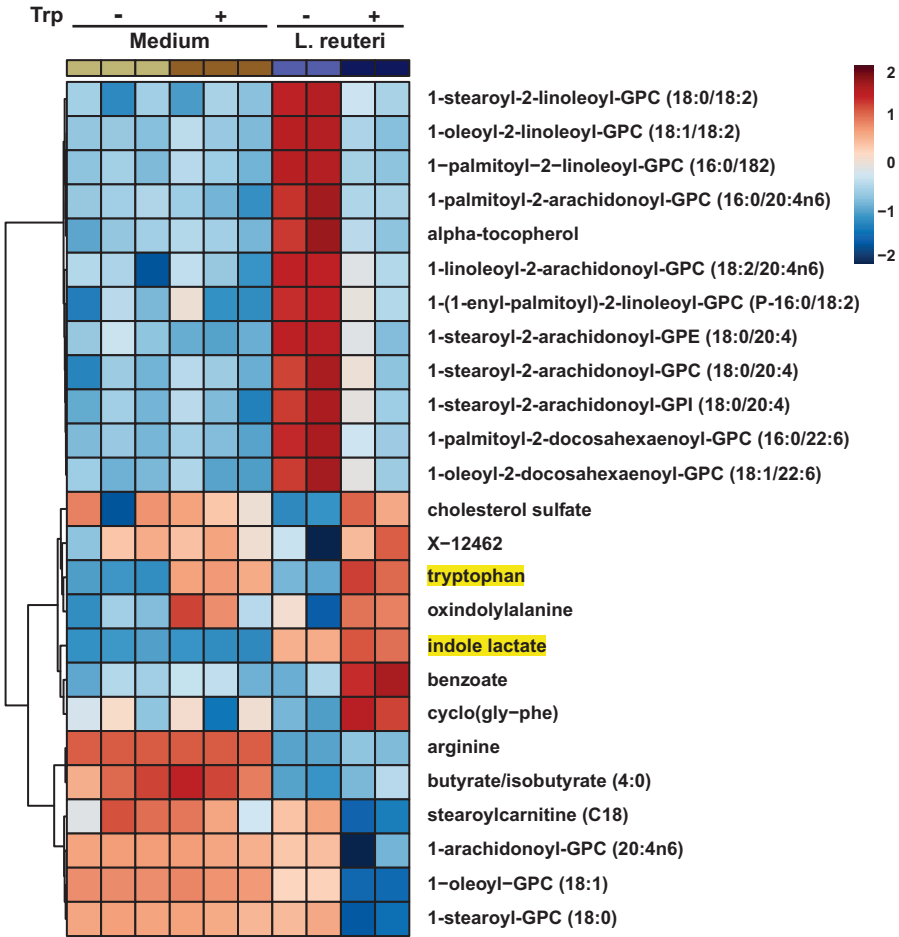

Supplement: Supplementary file 5 — Additional file 4: Figure S4. Tryptophan availability rewires L. reuteri metabolic output in monoculture. (A) Partial least-squares discriminate analysis (PLS-DA) and (B) hierarchical clustering by Euclidean distance using Ward’s linkage represented as a heatmap of total metabolites from basal BHI medium and L. reuteri monocultures with or without 1mM tryptophan supplementation. Heatmap reflects top 25 differentially abundant metabolites between L. reuteri monocultures with or without 1mM tryptophan and analyzed by t-test at a threshold of p≤0.05 to generate a subset of metabolites most influenced by L. reuteri tryptophan metabolism. The resulting list was analyzed in all four experimental groups (including media alone controls) by one-way ANOVA at p≤0.05 and represented as a heatmap. [file 40168_2022_1408_MOESM4_ESM.pdf]

Figure S5

A

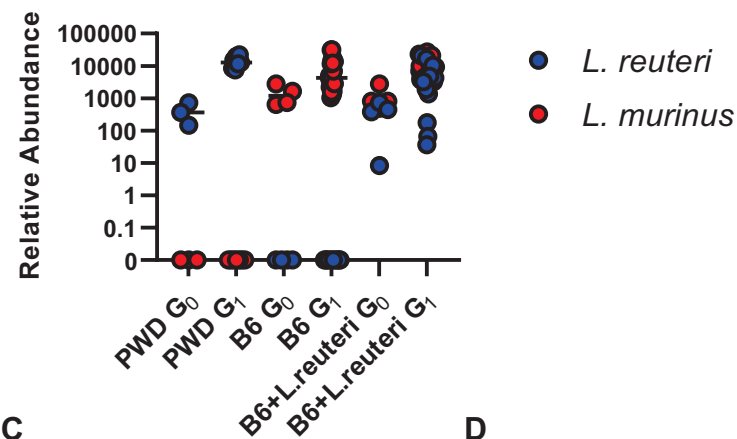

B

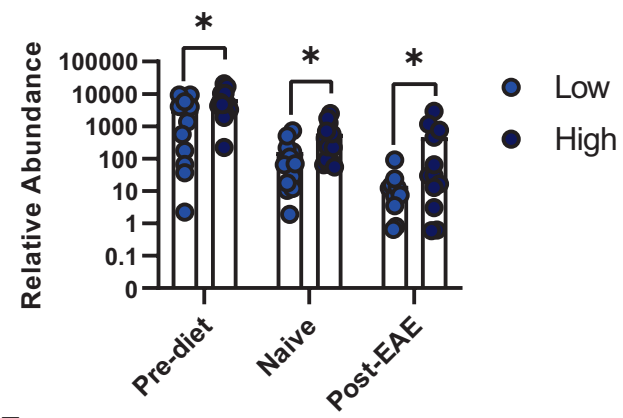

C

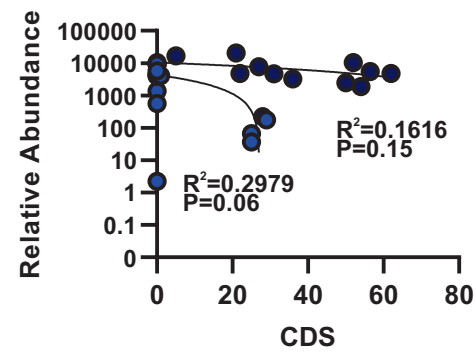

D

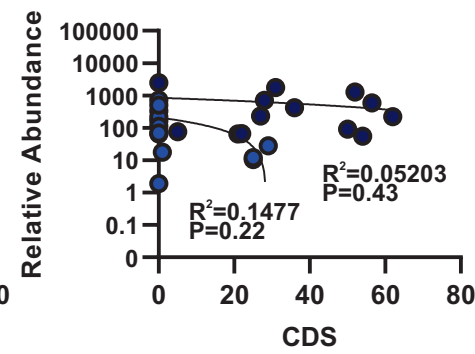

E

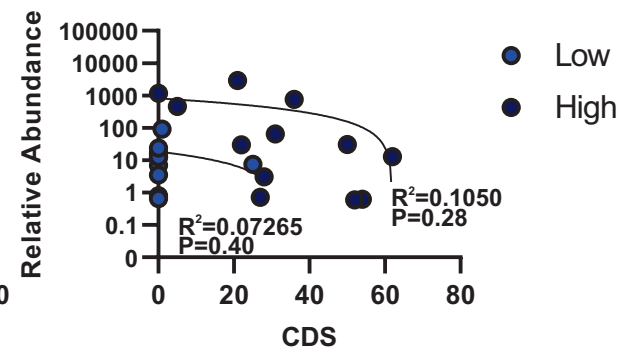

Supplement: Supplementary file 6 — Additional file 5: Figure S5. Abundance dynamics of L. reuteri in experimental breeding pairs and within dietary intervention studies. Founder G0 B6-GF mice were inoculated with cryopreserved donor cecal contents from PWD, B6, or B6 cecal contents supplemented with 109 CFU of L. reuteri and breeding pairs were established for vertical transmission to G1 offspring. Fecal samples were collected at 4-wks post-inoculation and in experimental offspring prior to dietary intervention (pre-diet), following 1-week of randomized diets (naive) and following a full 30-day disease course (post-EAE). Abundance of L. reuteri and L. murinus was determined by qPCR using species specific primers in (A) G0 breeders and G1 offspring and (B) throughout the course of dietary intervention. Relative abundance of L. reuteri at day 30 post EAE induction was correlated with disease severity as measured by cumulative disease score (CDS) using linear regression with the P value indicating significant deviation from a non-zero slop (i.e. significant correlation) for pre-diet (C), naive (D), and post-EAE (E) samples. [file 40168_2022_1408_MOESM5_ESM.pdf]

Figure S6

A

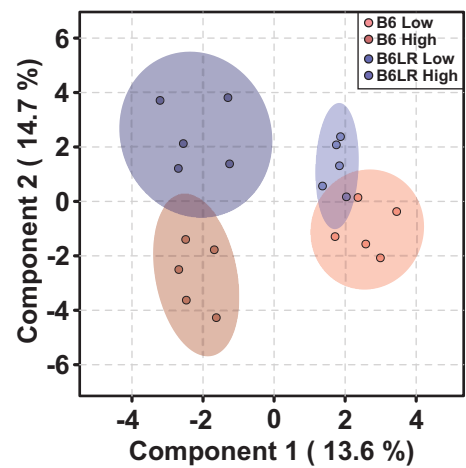

B

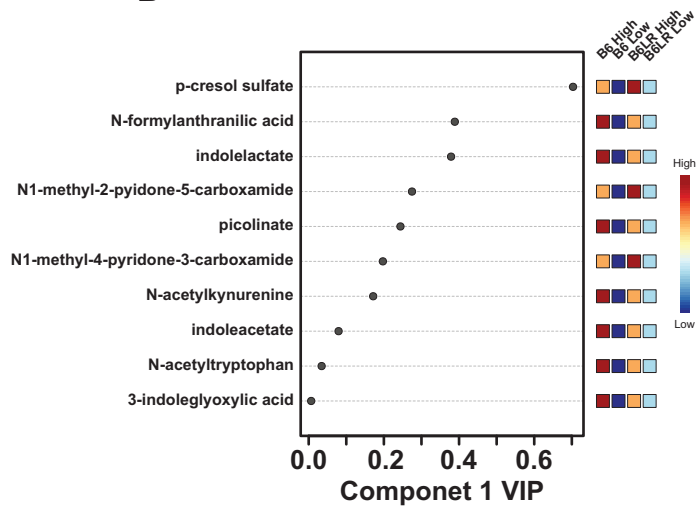

C

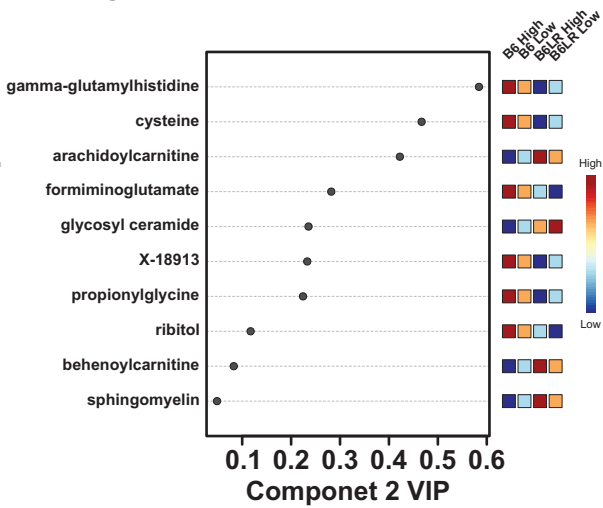

Supplement: Supplementary file 7 — Additional file 6: Figure S6. Microbiome and dietary signatures in pooled analysis of serum metabolomic data. Serum was collected from mice fed a low or high tryptophan diet colonized with either the B6 or B6+L. reuteri microbiome following a 30-day EAE course and analyzed via UPLC-MS/MS as outlined in (Fig. 4A and B). Data were analyzed for all four experimental groups: B6 or B6+L. reuteri colonized mice randomized to a low 0.02% or 0.8% high tryptophan diet. (A) Partial least squares-discriminant analysis (PLS-DA) of total metabolites. (B) Top 10 metabolites as variables of importance in the PLS-DA projection (VIP) along component 1 responsible for segregating samples by diet. (C) Top 10 metabolites as variables of importance in the PLS-DA projection (VIP) along component 2 responsible for segregating samples by microbiome. Statistical analysis is provided in Table S24. [file 40168_2022_1408_MOESM6_ESM.pdf]

Figure S7

A

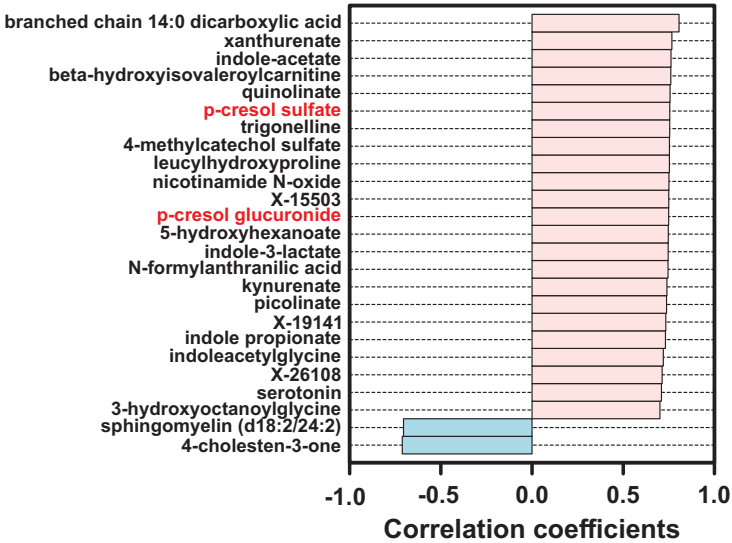

B

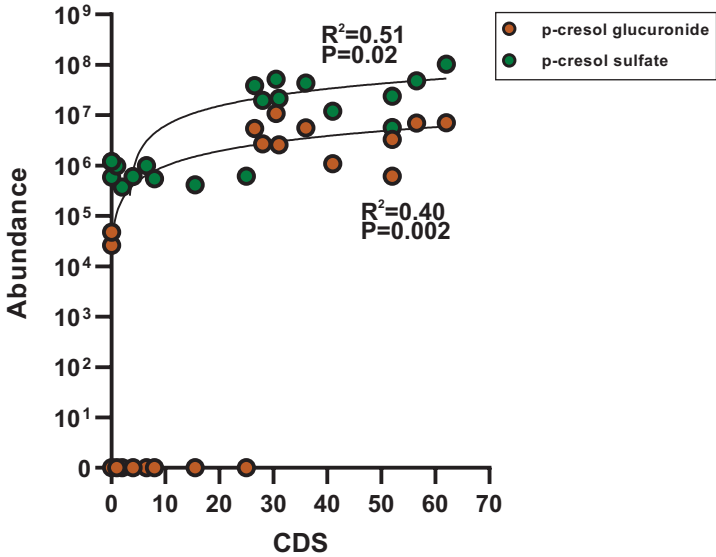

Supplement: Supplementary file 8 — Additional file 7: Figure S7. The abundance of p-cresol sulfate and p-cresol glucuronide correlate with disease severity. Serum was collected from mice fed a low or high tryptophan diet colonized with either the B6 or B6+L. reuteri microbiome following a 30-day EAE course and analyzed via UPLC-MS/MS as outlined in (Fig. 4A and B). Data were analyzed for all four experimental groups: B6 or B6+L. reuteri colonized mice randomized to a low 0.02% or 0.8% high tryptophan diet. (A) Top 25 metabolites correlating with disease severity as measured by cumulative disease score (CDS), the sum of all daily scores over a 30-day disease course. (B) X-Y scatter plot of p-cresol sulfate and p-cresol glucuronide abundance and CDS using linear regression, with the P value indicating significant deviation from a non-zero slope (i.e. significant correlation). A zero value was entered for p-cresol glucuronide abundance in low-tryptophan-fed mice when below the limit of detection. Statistical analysis is provided in Table S31. [file 40168_2022_1408_MOESM7_ESM.pdf]

**Figure S8**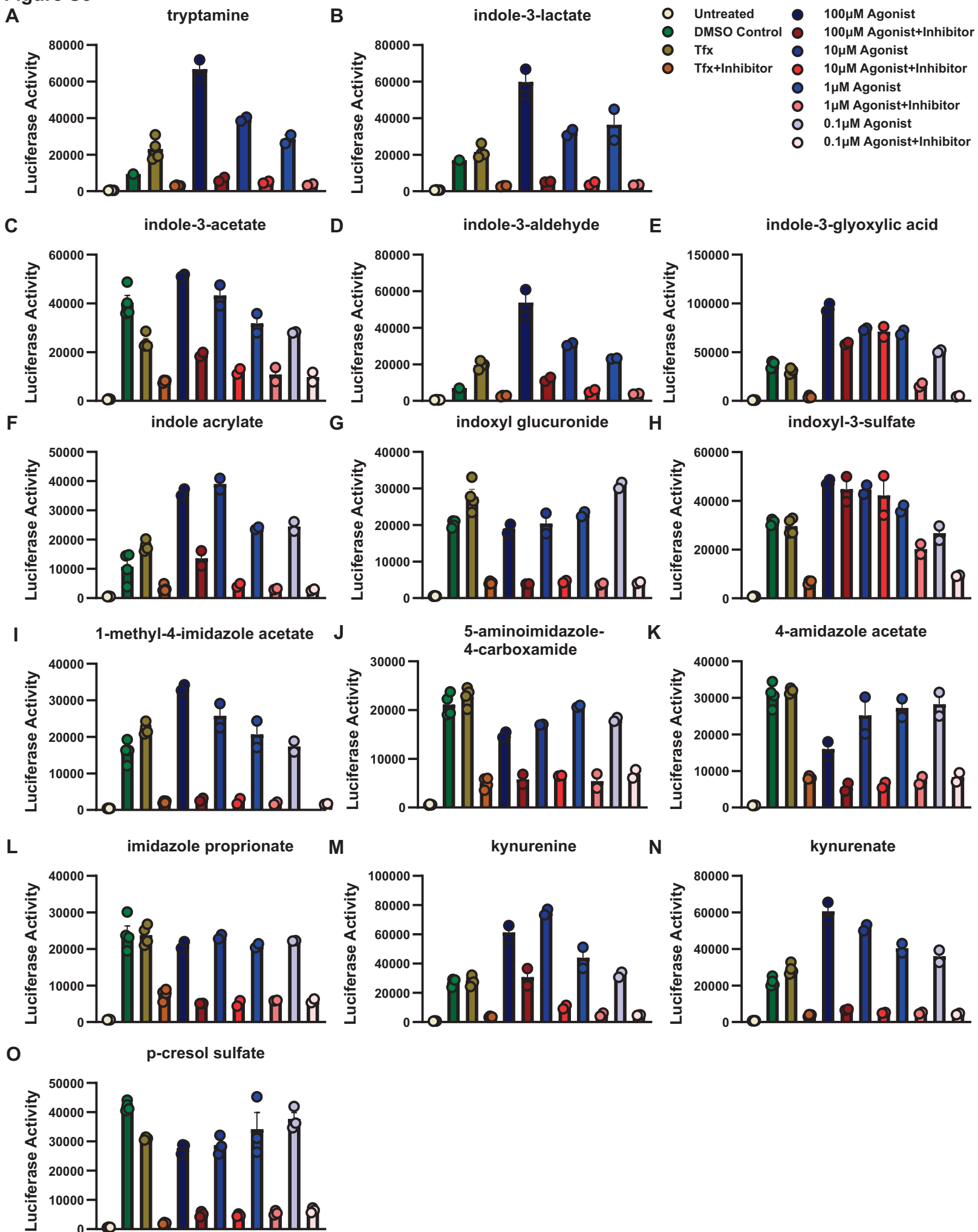

Supplement: Supplementary file 9 — Additional file 8: Figure S8. L. reuteri metabolites are ligands for the aryl hydrocarbon receptor. Selected metabolites identified in L. reuteri monoculture or serum by UPLC-MS/MS were analyzed in a cell-based luciferase assay for capacity to activate or inhibit the AhR at 100μM, 10μM, 1μM, and 0.1μM with or without 4hr pre-treatment with the AhR antagonist, CH223191 at 10μM. Transfection and DMSO vehicle controls are included for comparison. [file 40168_2022_1408_MOESM8_ESM.pdf]

Figure S9

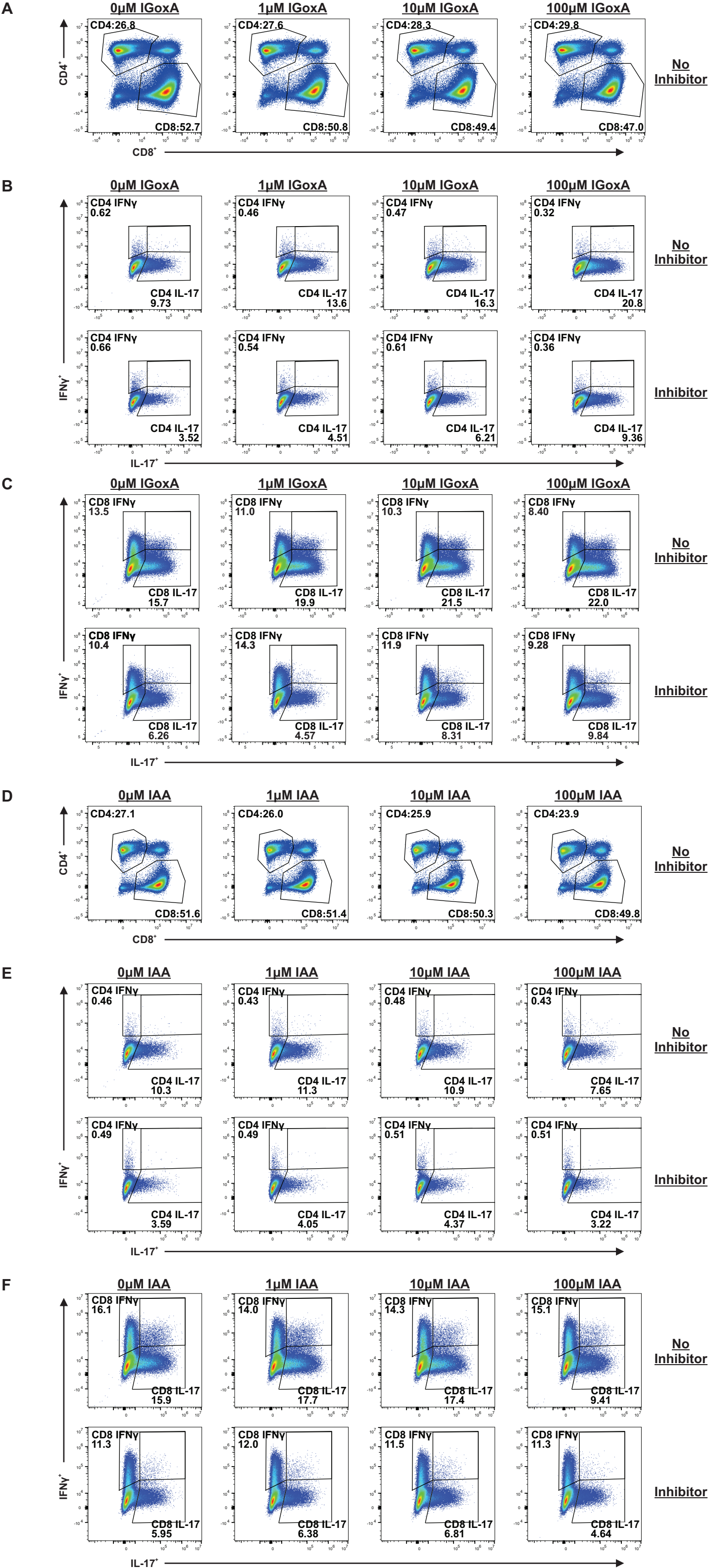

Supplement: Supplementary file 10 — Additional file 9: Figure S9. L. reuteri metabolites are sufficient to elicit a Th17 immune response. Splenocytes were differentiated under Th17 conditions without or without IGoxA, IAA, and/or AhR antagonist treatment at 0, 1, 10 or 100 μM followed by intracellular cytokine staining and flow cytometry. CD4+ and CD8+ T cell (A) frequency of total CD45+ T cells and IL-17 production (B and C) as frequency of each parent population in response to IGoxA treatment with and without AhR inhibitor. CD4+ and CD8+ T cell (D) frequency of total CD45+ T cells and IL-17 production (E and F) as frequency of each parent population in response to IAA treatment with and without AhR inhibitor. [file 40168_2022_1408_MOESM9_ESM.pdf]
